# B-1 derived anti-Thy-1 B cells in old aged mice develop lymphoma/leukemia with high expression of CD11b and Hamp2 that different from TCL1 transgenic mice

**DOI:** 10.1186/s12979-024-00415-6

**Published:** 2024-04-03

**Authors:** Kyoko Hayakawa, Yan Zhou, Susan A. Shinton

**Affiliations:** Fox Chase Cancer Center, 333 Cottman Ave., Philadelphia, PA 19111 USA

**Keywords:** B1, CLL/lymphoma, TC^+^ Tg, CD11b, Hamp2, V2-5

## Abstract

Human old aged unmutated chronic lymphocytic leukemia U-CLL are the TCL1^+^ZAP70^+^CD5^+^ B cells. Since CD5 makes the BCR signaling tolerance, ZAP70 increased in U-CLL not only TCL1^+^ alone. In mice, TCL1 (TCL1A) is the negative from neonate to old aged, as TC^–^. V_H_8-12/V_k_21-5 is the anti-thymocyte/Thy-1 autoreactive ATA B cell. When ATA μκTg generation in mice, ATA B cells are the neonate generated CD5^+^ B cells in B-1, and in the middle age, CD5^+^ can be down or continuously CD5^+^, then, old aged CLL/lymphoma generation with increased CD11b in TC^–^ZAP70^–^CD5^–^ or TC^–^ZAP70^+^CD5^+^. In this old aged TC^–^ATA B microarray analysis showed most similar to human CLL and U-CLL, and TC^–^ZAP70^+^CD5^+^ showed certain higher present as U-CLL. Original neonate ATA B cells showed with several genes down or further increase in old aged tumor, and old aged T-bet^+^CD11c^+^, CTNNB1^hi^, HMGB^hi^, CXCR4^hi^, DPP4^hi^ and decreased miR181b. These old aged increased genes and down miR181b are similar to human CLL. Also, in old age ATA B cell tumor, high CD38^++^CD44^++^, increased Ki67^+^ AID^+^, and decreased CD180^–^ miR15O^low^ are similar to U-CLL. In this old aged ATA B, increased TLR7,9 and Wnt10b. TC^+^Tg generated with ATAμκTg mice occurred middle age tumor as TC^+^ZAP70^–^CD5^+^ or TC^+^ZAP70^+^CD5^+^, with high NF-kB1, TLR4,6 and Wnt5b,6 without increased CD11b. Since neonatal state to age with TC^+^Tg continuously, middle age CLL/lymphoma generation is not similar to old aged generated, however, some increased in TC^+^ZAP70^+^ are similar to the old age TC^–^ ATA B tumor. Then, TC^–^ ATA B old age tumor showed some difference to human CLL. ATA B cells showed CD11b^+^CD22^++^, CD24 down, and hepcidin Hamp2^++^ with iron down. This mouse V8-12 similar to human V2-5, and V2-5 showed several cancers with macrophages/neutrophils generated hepcidin^+^ iron^low^ or some showed hepcidin^–^ iron^+^ with tumor, and mouse V8-12 with different V_k_19-17 generate MZ B cells strongly increased macrophage^++^ in old aged and generated intestine/colon tumor. Conclusion, neonate generated TC^–^ATA B1 cells in old aged tumor generation are CD11b^+^ in the leukemia CLL together with lymphoma cancer with hepcidin-related Hamp2^++^ in B-1 cell generation to control iron.

**Supplementary Information:**

The online version contains supplementary material available at 10.1186/s12979-024-00415-6.

## Introduction

In human and mice, the Lin28b^+^Let7^–^ axis plays a major role in fetal/neonatal development, whereas Lin28^–^Let7^+^ cells are more important in adults [1]. In old age, function of the adaptive immune system is diminished [2], whereas increased function of the innate immune system is more important such as myeloid cell differentiation and fetal/neonatal origin B-1 B cells. In old age in human, chronic lymphocyte leukemia (CLL) is the most common type of leukemia in Western countries. Human CLL is CD5^+^ B cells and is often found with mutated IGH gene (M-CLL) which has a significantly better overall survival than patients with unmutated (U-CLL) [3, 4]. U-CLL often found as IGHV1-69, which can originate in the fetal/neonatal stage from cord blood [5]. Overall, CLL is lower in Africa than in Western countries, however CLL originating in Africa showed higher incidence of U-CLL with V1-69 than M-CLL [6]. Since original fetal/neonate generation levels and adult B and T cell interaction with containing original B-1 cells, early origin background is important to old age in U-CLL.

In mice, CLL is rarely observed. However, NZB mice show CLL generation [7] and NZB cells showed high NTA(natural thymocytotoxic autoantibodies) expression associated with CD5^+^ B cells [8]. Most mice generate NTA expression, and SM/J and NZB mice express higher NTA than BALB/c and C57BL/6 [9]. We first found NTA expression in SM/J mice at the immature T cell stage drives positive selection of B cells with high expression of V_H_8-12/V_k_21-5 BCR, which showed anti-thymocyte/Thy-1 autoreactive (ATA) B cells [10]. We then, generated ATAμκTg in C.B17 mice (BALB/c–Igh^b^), in which ATA B (Igh^a^) cells are the largely product of fetal/neonatal B cell development (B-1) as CD5^+^B1a, normally constituting B-1 cell subset in adult, as B1 B cells [11]. In adult B cell development (B-2) from BM, the expression of V_H_8-12/V_k_21-5 BCR drives negative selection, resulting in developmental arrest [11]. Then, these B-1 origin V_H_8-12/V_k_21-5 ATA B cells can generate leukemia/lymphoma in old aged with increased expression of CD11b [12, 13]. This paper now showed with CD11b-CD22^++^, and also found that very high level of hepcidin-Hamp2 in ATA B cells in old age.

Mouse ATA B leukemia/lymphoma are TC^–^, α-Thy-1 ATA B cell, and Hamp2^++^. These differences between human versus mice. In human CLL, TCL1^+^ positive and U-CLL CD5^+^ B cells with ZAP70^+^. TCL1 (TCL1A,B) are expressed in the embryonic stage in mice and human, originally [14, 15]. Then, in mice, TCL1 (TCL1A) is down regulated in fetal liver and then negative in neonate, becoming TCL1^–^ through adulthood [16]. In human, Pro-B, Pre-B, and immature B cells are TCL1^+^, and in spleen, TCL1 is still positive in immature B cells, then decreased in mature B cells to minimal levels [17]. Then, vascuration made to increase TCL1 in human cells and original B-1 unmutated CD5^+^ B cells can increased with TC^+^ [18, 19], also adult B cells can generated mutated CD5^+^ B cells with TC^+^. CD5 binds to SHR-1 related to the BCR, CD5 makes the BCR signaling tolerance [20, 21]. Thus, not only TCL1^+^ which BCR signaling hyperresponsive [22], ZAP70^+^ made more responses in human U-CLL for further response with autoimmune predisposition, showed TC^+^ZAP70^+^CD5^+^ [23]. In aged mice, B cells are continuously TCL1^–^ (TC^–^). Some original TC^–^ CD5^+^ B1a cells can downregulate CD5 in aged [24, 25]. Thus, ATAμκTg B lymphoma/leukemia can be generated from TC^–^ZAP70^–^CD5^–^, or TC^–^ZAP70^+^CD5^+^(or CD5^–^) B1 cells. We previously observed that transgenic expression of TCL1^+^(TC^+^)Tg in ATA B cells induced CLL/leukemia in middle aged mice [24], which originated from TC^+^ZAP70^–^CD5^+^ or TC^+^ZAP70^+^CD5^+^(or CD5^–^) B1 cells. Since human U-CLL is TC^+^ZAP70^+^CD5^+^, we further compared TC^–^ and TC^+^Tg ATAμκTg mice by microarray analysis, to determine why TC^+^Tg mice showed lymphoma/leukemia development in middle age instead of old aged. In this paper, we found that RagKO ThyKO ATAμκTg mice which were TC^–^CD5^–^Thy-1^–^ and lacked mature T and B cells, showed high ZAP70^+^ B1 cells with early state of CLL/lymphoma. In addition to being ZAP70^+^, we found old aged generated ATA B TC^–^ZAP70^–^CD5^–^ B1 cells with CD11b^++^, most of which were similar to old aged human TC^+^CLL/U-CLL cells.

Thy-1(CD90) is heavily N-glycosylated, glycophosphatidylinositol anchored cell surface protein. Thy-1 controls inflammatory cell recruitment in human and mice [26, 27], and Thy-1 plays an important role in the initial stages of virus infection [26]. Fetal hepatocytes are important in Thy-1^+^ human and mice [28, 29]. However, human Thy-1 is not generate in T cells begining in thymus, which differs from mouse T cells [30]. Thus, the original B-1 cells can generate ATA B cells in the thymus of mice [10] but not in human. However, Thy-1 is continuously important in human and mice with Thy-1 expressed on nerve cells (neuron) in brain, fibroblast, endothelial cells, monocytes, myeloid cells, and kidney [30–32], and serum Thy-1 is present. Thy-1 moves to endothelial cell to control the initial state of virus infection, and CD11b (Mac-1) helps to Thy-1 activated to endothelial move [33]. In humans, this CD11b is mostly expressed by neutrophils/monocytes (CD11b^+^Gr-1^+^) to move Thy-1 to endothelial tissues [33, 34]. Since Thy-1 is important in the nervous system of mice and human, when lacking Thy-1 in brain, strongly inhibited in the dentate gyrus [35]. Mouse thymocyte autoantibodies from ATA B1 cells react with Thy-1 in thymus, then original peritoneal cavity pB1a with increased CD11b but not in spleen B1a, and in old age CD11b^+^ATA B cells, and then, ATA B cells exhibit multiple specificites [36].

Mouse ATA B cell lymphoma/leukemia express high levels of Hamp2^++^ which is hepcidin. Hepcidin is found as Hamp1 and Hamp2 in mice, and human hepcidin is similar to mouse Hamp1 [37]. Hepcidin is an innate antimicrobial agent that is induced by most invasive bacteria and also by virus, then limits bacterial proliferation by reducing iron such as in plasma and extracellular fluids, and kills bacteria [38]. Hamp2 is more reactive as an antibacterial and antivirus activity than Hamp1 [39, 40]. The most important function of hepcidin (also mouse both Hamp1 and Hamp2) is to regulate iron [41]. Hepcidin is mostly produced by macrophages/monocytes, and TLR induced hepcidin by T cells and B cells [42]. ATA B cell tumors in old aged show high Hamp2 expression and decreased iron. This mouse ATA V8-12 V_H_ is related to human V2-5 which is also cancer related with hepcidin^+^ or iron^+^ (Fig. 5D). V_H_8-12/V_K_21-5 generated in ATA B1 cells, and previously shown V_H_8-12/V_k_19-17 generated in MZ B cells. Here, we show that this MZ B cells induces the generation of high macrophages in spleen with intestine/colon tumor in old age. Thus, expression of V8-12 in mice is related to B-1 cells with CLL, and cancers with Hamp2^++^ iron^low^ in old age, and V8-12 B-2 MZ B cells increased generation of macrophages with tumor generation in old age.

## Results and discussion

### CD11b^++^ B CLL/lymphoma develops in old age of mice from B-1 origin V_H_8-12/V_k_21-5 ATA Tg-expressing cells. Mouse V8-12 homologous to human V2-5

**Fig. 1:**
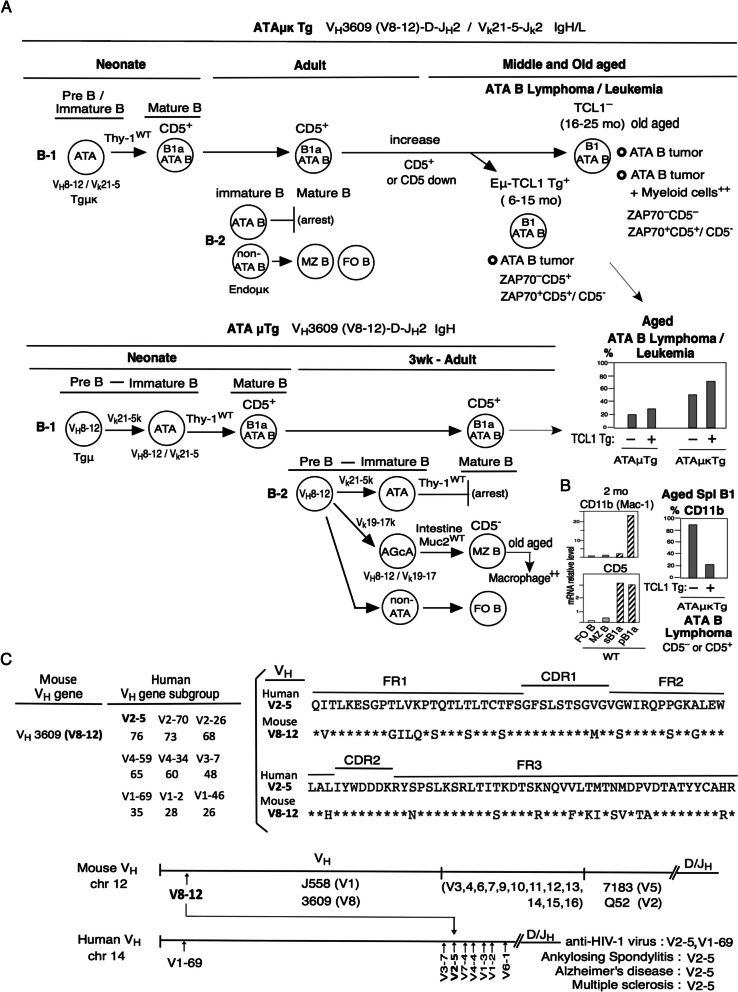
Summary of B-1 ATA B and B-2 AGcA cell generation, and mouse V_H_V8-12 similar to human V2-5. **A** and **B** V_H_8-12/V_k_21 ATAμκ Tg mice generate B-1 ATA B cells in fetal/neonate stage and generate CD5^+^B cells as B1a, differently, negative selection (arrest) occurs during adult B-2 development. B-1 ATA B cells increase in PBL in middle age, and CD5 down can occur or continuous CD5^+^. In old age CLL/lymphoma generation, CD5^–^ cells mostly not increased ZAP70, and increased ZAP70 with CD5^+^ ATA B cells. TC^–^ (CD5^–^ and CD5^+^) increased CD11b (61/73=84%) in CLL/lymphoma, in contrast, Eu-TCL1^+^ Tg cells generated at middle aged CLL/lymphoma are few increased CD11b (13/60=22%) [13]. V_H_8-12 ATAμTg mice can generate V_H_8-12/V_k_21 B1a and generate CLL/lymphoma in old age (low), and V_H_8-12/V_k_19 generate MZ B cells and V_H_8-12/V_k_19μκTg mice generated at 3 wk MZ B cells in B-2 and in old aged generate macrophage^++^. **C** Mouse V8-12 VH check with human VHs from UniProt and IMGT. Mouse V8-12 V_H_ gene is similar to human V2-5

As previously shown (Fig. 1A), expression of V_H_3609(V_H_8-12)/V_k_21-5 transgene (ATAμκ Tg) in the PreB/immature stage drives positive V_H_8-12/V_k_21-5 selection in B-1 cells through a process involving Arid3a-driven fetal/neonatal B-1 cell development to induce mature CD5^+^ B1a cells and constitute B1a cells in adults [43]. In adult B cell development from BM (B-2), the expression of V_H_8-12/V_k_21-5 BCR in immature B cells drives negative selection at the mature B cell stage, resulting in arrest [11]. As shown in Figure S1A, at the 2 mo stage of ATA B cell development, the PerC, mLN, and intestinal PP cell populations were mature B1a, but BM and Spl cells were dominantly immature. ATA B cells in PBL are immature at 2 mo, but an increased fraction of mature are at 8 mo, which corresponds with increased serum IgM, IgG1, IgG3 by 12 mo of age, whereas IgA is already high by 2 mo. However, when Pla2g2a was deleted at the neonate stage, the generation of mature ATA B cells was reduced including through 14 mo of age [12] (Fig. S1A). Thus, ATAμκTg B cells originally generate neonatal ATA B cells pB1a increase in PBL as mature in middle age and old age. The original anti-thymocyte/Thy-1 autoreactive mAbs from these B cells are polyspecific and can also bind multiple tumor antigens [36].

CD5 recruits the SHP-1 protein tyrosine phosphatase to inhibit BCR signaling [20, 21]. Thus, CD5^**+**^ B1a cells have more anti-apoptotic signals, serve as a source of autoantibodies, and are considered innate lymphocytes [44]. The Lyn-CD22-SHP-1 pathway promotes BCR tolerance [45]. CD11b also regulates BCR by binding to CD22 [46]. Macrophages in peritoneal cavity can induce B1a cells into PerC [47], which are mainly CD5^+^CD11b^+^. When these PerC B1a cells move to spleen, complement component C3 in spleen can decrease CD11b expression as CD11b^–^ [48], but CD5 expression, thus autoantibody production are retained. However, LPS or CpG can downreguate CD5 in adult B1 cells to generate CD5^–^ B1 cells [24] (Fig. S1B). These CD5^–^CD11b^–^ B1 cells in spleen exhibit strong BCR signaling. In middle age, both TCL1^–^ (TC^–^) and TCL1^+^ Tg (TC^+^ Tg) ATA B cells can become CD5^+^ or CD5^–^, although TC^–^ ATA B cell tumors are most CD5^–^, TC^+^ Tg ATA B cells are mostly continuously CD5^+^ (Fig. S1B). The TC^+^ Tg ATA B cells generate lymphoma/leukemia at middle age [24], whereas TC^–^ ATA B cell tumors are in old age. In older humans, TC^+^ CLL can develop, in which most tumor cells are CD5^+^. However, 7–20% human CLL B cells are CD5^–^ [49], and these human CD5^–^ B cells induce splenomegaly [50], which is consistent with increased splenic CD5^–^ B1 cells in the mice.

Mouse TC^+^ Tg expressing ATA B cells generate slightly more lymphoma/leukemia in middle age than TC^–^ cells (Fig. 1A). However, TC^–^ ATA B tumor cells in old age showed the recovery of increased CD11b expression (Fig. 1B) [13] and react with CD22 (shown in Fig. 5C). At 2 mo age, CD11b^–^ splenic B1a cells stimulated with CpG increased expression of CD11b (or LPS, but low), and the combination of CpG with IL-10 further increased CD11b [13] (Fig. S1C). As previously showed (in Figure S1C), splenic aMyIIA V_H_Q52 B1 cells and aPtC V_H_11 B1 cells (V_H_ knock in) also increased CD11b expression in old age [13, 25]. PerC B1a cells originate as CD5^+^CD11b^+^, then become CD5^+^CD11b^–^ upon migration to spleen, then further to CD5^–^CD11b^–^, and middle aged CD11b increased, then, CD5^–^ CD11b^++^ tumor cells develop in spleen from ATA B cells in old aged mice. In human TC^+^CD5^+^ CLL, 18% of tumor cells are CD11b^+^ but most are CD11b^–^ [51], which differs from the TC^–^ mouse. Some mouse TC^–^ ATA B cells progress to lymphoma/leukemia at old age together with increased myeloid cells [13] (Figs. 1A and 5E), which is consistent with some human CLLs that develop with myeloid cells [52, 53].

In ATAμTg (V_H_8-12/D/J_h_2) mice, CD5^+^ B1a ATA cells generated lymphoma/leukemia in old age, but at a lower rate than ATAμκTg mice [24] (Fig. 1A). Mice co-expressing V_H_8-12 and V_k_19-17/J_k_1 (as V_H_8-12/V_k_19-17) generated strong MZ B cell development, and some V_H_8-12 FO B cells were also generated [54]. The V_H_8-12/V_k_19-17 BCR is a natural anti-intestinal goblet cell work (AGcA), V_H_8-12/V_k_19-17μκTg mice strongly generated MZ B cells [54], and some MZ B AGcA cell-derived tumors in old age (Fig. S3). MZ B cells are the major constituent of the marginal zone, together with myeloid, dendritic, and stromal cells [55]. Generally, macrophage/neutrophils CD11b^+^Gr-1^+^ cells generate hepcidin to control iron [56], and also hepcidin^low^ iron^+^. Then, old aged in AGcA Tg mice generated high macrophage (CD11b^+^Gr-1^+^) (Fig. 1A), and as showed in Fig. 5E and Fig. S3, this macrophages made strong intestine/cold tumor in old aged, not hepcidin related positive, rather iron^+^ increased.

The mouse V_H_V8-12 BCR is homologous to human V2-5 (Fig. 1C). Human V1-69 is expressed in unmutated CLL (U-CLL), but V2-5 is low in CLL [57, 58]. Although human V2-5 is very low in cord blood [59], in depth analysis showed V4-59, V4-34, and V2-5 in cord blood [60], suggesting that V2-5 reacts in neonates. Importantly, it is also well known that HIV-1 (human immunodeficiency virus) is associated with increased V2-5 (2F5) and V1-69 (4E10) in humans [61] (Fig. 1C). V2-5 is also found in Ankylosing Spondylitis (AS) [62] and Alzheimer’s disease (AD) [63, 64]. AD was also found to be associated with mouse V8-12 as human V2-5 [65]. Then, it has long been known that dominant V2 (most V2-5) expression is associated with increased susceptibility of multiple sclerosis (MS) as brain-gut axis with iron^+^ [66]. Several cancers in human have used V2-5 as listed in Fig. 5D. V2-5 immunoglobulin are variable, most hepcidin^+^ and some showed hepcidin^low^ iron^+^. Thus, in this paper, first mouse TC^–^ ATAμκ Tg lymphoma/leukemia compared to human old aged TC^+^ CLL/U-CLL, and TC^+^ ATAμk Tg middle aged may difference from TC^–^ in old aged. Then, since TC^–^ ATAμκ Tg B cells found Hamp2^++^ generated in old age different from human CLL, thus, checked with human V2-5 positive cancers with hepcidin^+^ or some iron^+^ lists (Figs. 4 and 5).

### Rag1KO Thy1KO ATAμκTg mice generate ZAP70^+^CD5^–^ATA B-CLL/lymphoma at early mature and middle age

**Fig. 2:**
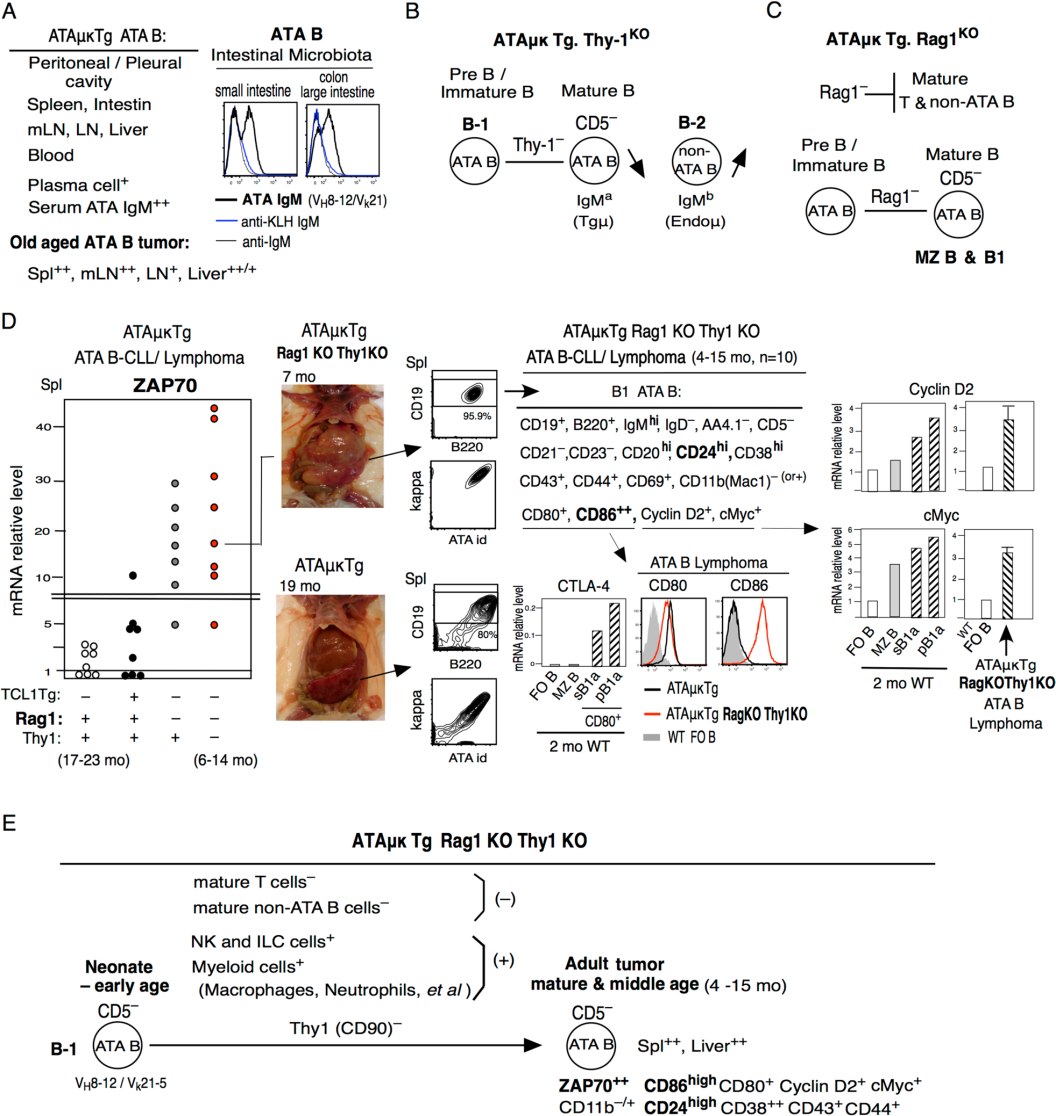
Rag1KOThy1KO ATAμκTg mice generate mature &middle age ATA B tumor. **A** Summary of ATAμκTg ATA B generation and old aged ATA B tumor place. ATA B IgM react with intestinal microbiota, and higher in colon, a-IgM is the control. **B** and **C** Summary of ATAμκTg with Thy-1^KO^ or Rag1^KO^. Thy-1^KO^ showed decreased B-1 ATA B cells with CD5^–^, rather B-2 non-ATA B cells increase. Rag1^KO^ showed increased MZ B and B-1 ATA B, and both not generated CD5. **D** and **E** ZAP70 levels in ATA B-cell/lymphoma generation in TC^–^, TC^+^, TC^–^Rag1^KO^, and TC^–^Rag1^KO^Thy-1^KO^ mouse levels with negative FO B as set to 1.0. TC^–^ATAμκTg Rag1^KO^Thy-1^KO^ATA B mice highest ZAP70, and generated early (mature & middle) age ATA B-cell/lymphoma generation. High CD86 and high CD24 are different with old age most TC^–^ZAP70^–^CD5^–^ ATAμκTg mice, but cyclin D2 and c-Myc are continually high as TC^–^ATAμκTg mice

Previously shown Fig. 2A,B,C. Neonate ATAμκTg mature ATA B cells arise first in the peritoneal cavity (PerC) with the support of macrophages [47] and pleural cavity, and present in spleen, intestine, mLN, LN, liver, and blood of aged mice, which also exhibit strong ATA IgM in serum and plasma cells [11]. ATA B cell tumors in old aged mice are Spl^++^, mLN^++^, LN^+^, and Liver^++/+^ [13]. In intestinal microbiota, ATA IgM is present in intestine and highest in colon (Fig. 2A). At 2 mo, mature B1 cells are already present in PerC, mLN, and intestinal Peyer’s patches (PPs) (Fig. S1). In contrast, as previously shown, in Thy-1KO ATAμκTg mice, the original ATA Tg does not generate CD5^+^ B1 cells, rather, B-2 non-ATA B cells are increased [55] (Fig. 2B). Rag1KO ATAμκTg mice engineered, mature T and non-ATA B cells were not generated, however, MZ ATA B cells are strongly generated and also ATA B1 cells, both of which are CD5^–^ [55] (Fig. 2C).

ATAμκTg mice with both Rag1KO and ThyKO background also generated B1-CLL/lymphoma at mature and middle age without CD5 expression, and these tumors expressed higher levels of ZAP70 than TC^–^ or TC^+^ ATAμκ Tg mice, which are most CD11b^–^ (75%) (Fig. 2D). These tumors express cyclin D2, cMyc, and CTLA-4 as high as originally generated initial B1 cells. Although CTLA-4 is associated with higher CD80 than CD86 in normal mouse B1a cells [67], ZAP70^hi^ RagKOThy1KO mice showed higher CD86 than CD80 and also increased CD24 expression. Mature T cells and non-ATA B cells are absent in Rag1KO mice, but NK and ILC cells and myeloid cells are continuously present. TC^–^CD5^–^B1 ATA B cells with most CD11b^–^ are generated with ZAP70^++^ at early age including BCR signaling and found in high numbers in spleen and liver but not in mLN. These early RagKOThy1KO ATA B cell tumors are different from Rag^+^Thy1^+^ATA B cells, which are generated at the neonate stage and adult animals controlling tumor development with the presence of mature B-2 and T cells together with Thy-1^+^, but tumors still develop in old age.

### Decreased Nod1, IL5R, CD1d, and increased CD11b in the B1 cells in old aged CLL

**Fig. 3:**
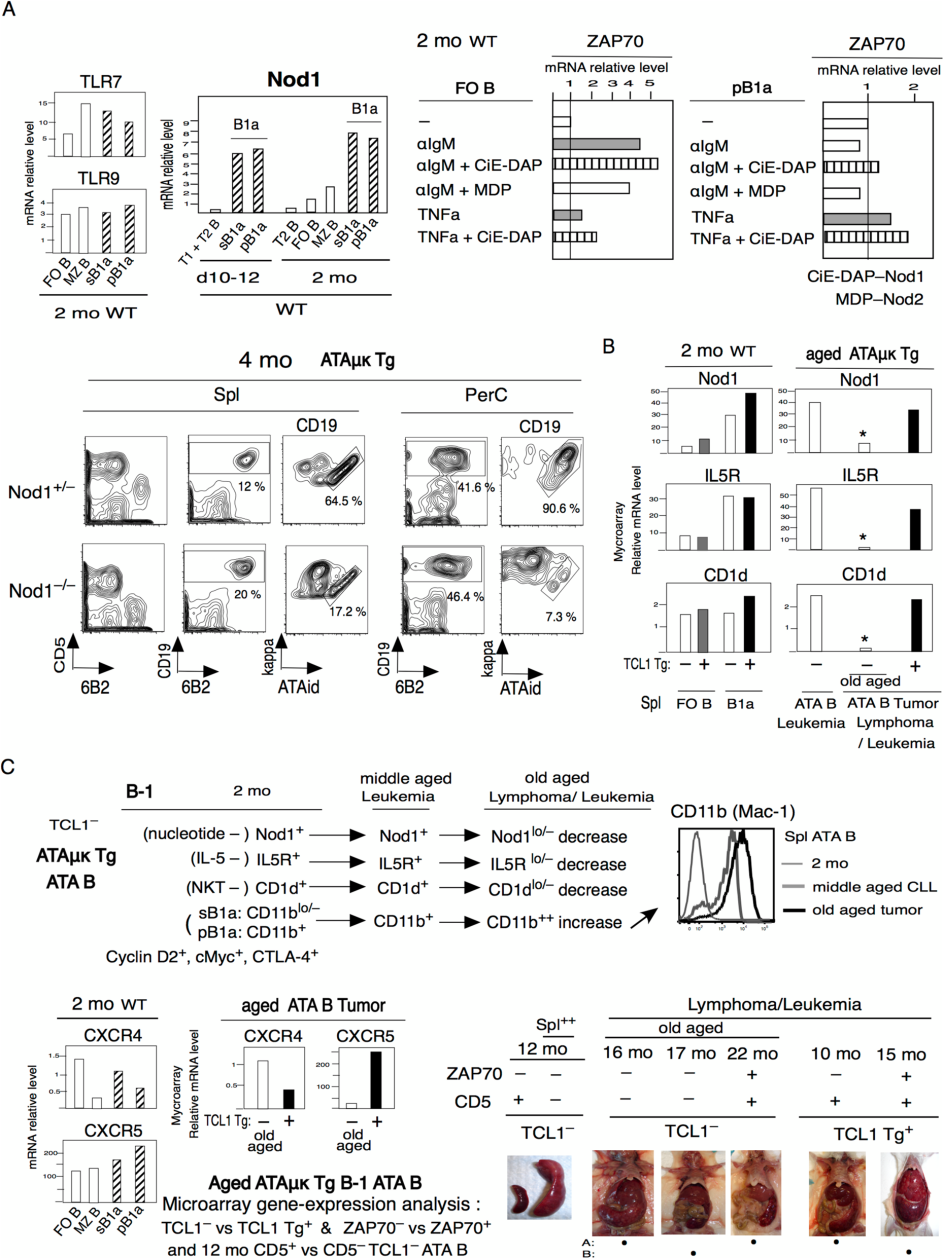
Nod1 is important in early B1a cells and Nod1, IL-5R, CD1d decrease in old aged and increased CD11b and CXCR4, different from middle aged TC^+^Tg ATA B cells. **A** Nod1 high in neonate and adult B1a. 2 mo FO B versus pB1a for ZAP70 generation after 20 hour later by anti-IgM without or with CiE–DAP (Nod1) or MDF (Nod2), or TNFa without or with CiE-DAP. ZAP70^hi^ in a-IgM is only FO B cells not CD5^+^pB1a, but Nod1 add increased ZAP70. Down: 4 mo ATAμκ Tg mice with NOD1^+/–^ or NOD1^–/–^. ATAid is originally generated V_H_8-12/V_K_21-5, then, low ATA B cells in Nod1^–/–^. Thus, Nod1 is important. **B** Nod1, IL-5R, CD1d comparison between TC^–^ versus TC^+^Tg ATAμκTg, in 2 mo and meddle aged leukemia CLL and old aged TC^–^ CLL/lymphoma. Old aged TC^–^ tumor showed down these lists, not middle aged TC^–^ and TC^+^Tg. **C** Conclution of TC^–^ATAμκTg ATA B in 2 mo verus old age tumor stage in spleen, with increased CD11b in TC^–^ ATA B in flow cytometry analysis. Down: In microarray analysis, TC^–^ old age ATA B cells are CXCR4 increase versus CXCR5 down, different compared with TC^+^Tg. Down right: Several TC^–^ versus TC^+^Tg ATAuk Tg lymphoma/leukemia pictures used for Fig. 4: 16 mo and 17 mo TC^–^ZAP70^–^CD5^–^ (Spl^++^, mLM^++^, LN^+^), 22 mo TC^–^ZAP70^+^CD5^+^ (Spl^++^, colon^++^, LN^+^), 10 mo TC^+^ZAP70^–^CD5^+^ (Spl^++^, mLN^+^, PerC^++^), 16 mo TC^+^ZAP70^+^CD5^+^ (Spl^++,^ Liver^++^, PerC^++^), and 12 mo samples Fig. 5

Neonate-generated B1a cells are known to express high levels of Nod1 and remain continuously Nod1^+^, as compared to the low levels in B-2 generated cells [68] (Fig. 3A). These B1a cells are also slightly higher in TLR7 and TLR9 than FO B at 2 mo (Fig. 3A). When FO B and pB1a cells were stimulated, FO B cells were found to express high levels of ZAP70 mRNA by anti-IgM, but this did not occur in B1a cells (Fig. 3A) since CD5 expression in B1a does not require BCR signaling. However, combining CiE-DAP for Nod1 reaction with anti-IgM increased ZAP70 expression, but not Nod2, in B1a cells also in FO B. Treatment with TNFa increased ZAP70 expression in both B1a and FO B, and further increases ZAP70 when TNFa was combined together with Nod1 expression. Nod1 is important for B1a cell increase, since at 4 mo, Nod1^–/–^ are low ATA B cells in ATAμκTg mice. In addition to Nod1, IL5 is also required for B1a cell generation and maintenance in contrast to FO B cells [69], and expression of CD1d is essential for NKT cell reactivity are similar between B1a and FO B cells as previously shown [12]. ATA B CLL generated at middle age is continuously Nod1^+^, IL5R^+^ and CD1d^+^, also in the presence of TC^+^Tg. In TC^–^ ATA B cell tumors, expressions of these decreased in old aged mice [12] ( Fig. 3B). Compared, CD11b expression is increased on CLL developing in middle aged spleen, and further increased on tumors at old age [12, 13] (Fig. 3C). Original B1a cells at 2 mo express more CXCR5 than CXCR4, whereas TC^–^ATA B cell tumors at old age showed decreased CXCR5 and increased CXCR4, as compared to TC^+^Tg mice (Fig. 3C). Similarly, in aged humans, CLL that arises CXCR4 expression higher than CXCR5 [70].

**Fig. 4:**
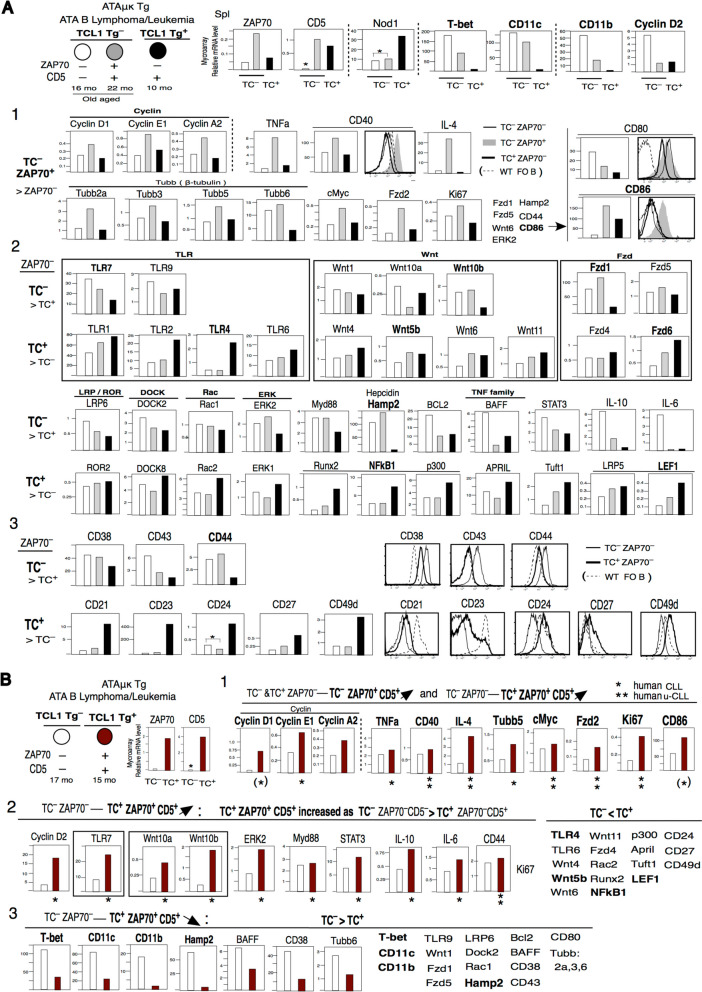
Microarray mRNA analysis by ATA B lymphoma/leukemia. TC^–^ZAP70^–^CD5^–^, TC^–^ZAP70^+^CD5^+^ vs TC^+^ZAP70^–^CD5^+^ and TC^–^ZAP70^–^CD5^–^ vs TC^+^ZAP70^+^CD5^+^. ATA B in spleen. **A** A1: Higher of TC^–^ZAP70^+^CD5^+^ list. A2,3: higher of TC^–^ZAP70^–^ than TC^+^ZAP70^–^CD5^+^ (TC^–^ >TC^+^) and higher TC^+^ZAP70^–^CD5^+^ than TC^–^ (TC^+^ > TC^–^) list. A3: add flow cytometry analysis of TC^–^ versus TC^+^ ATA B lymphoma/leukemia together with WT FO B cell. **B** B1: TC^+^ZAP70^+^CD5^+^ similar to high TC^–^ZAP^+^CD5^–^ cells. B2: TC^+^ZAP70^+^CD5^+^ also several high TC^–^ZAP70^–^CD5^–^ cells. TC^+^>TC^–^ are not listed by genes TC^+^ZAP70^+^CD5^+^ decreased to TC^–^. B3: TC^+^ZAP70^+^CD5^+^ cells and also TC^+^ZAP70^–^CD5^+^ are not increased to old aged TC^–^ cells (ZAP70^–^CD5^–^, ZAP70^+^CD5^+^) as TC^–^ > TC^+^

**Fig. 5:**
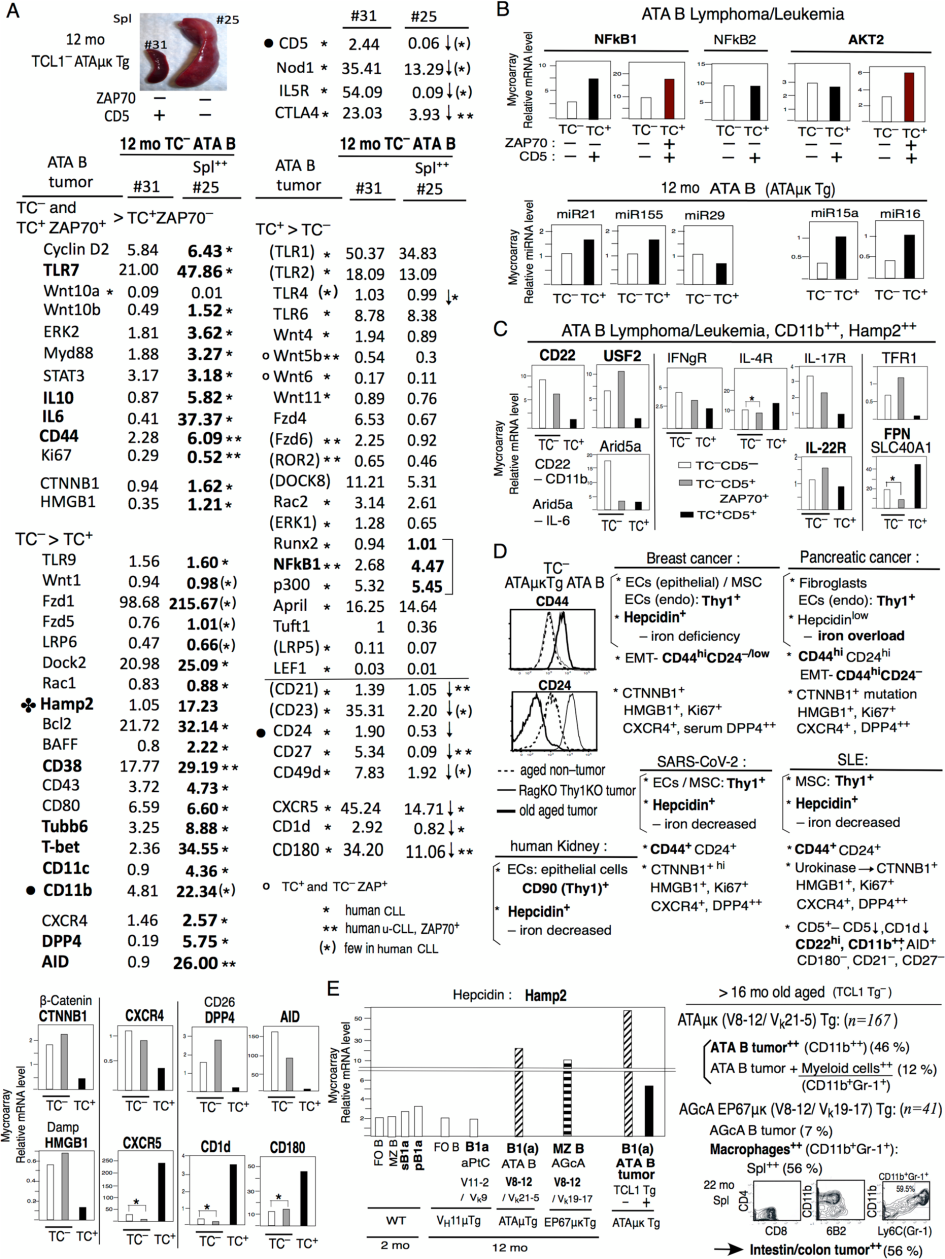
12 mo TC^–^CD5 down ATA B spleen^++^ cells similar to old aged TC^–^ATA B lymphoma/leukemia. TC^+^ Tg ATA B lymphoma/leukemia are NF-kB1 high. Old aged TC^–^ ATA B are CD11b^++^CD22^++^, CD24^low^, and Hamp2^++^ generation, and V8-12 with V_k_19 AGcA MZ B are 12 mo Hamp2^+^ and old age generated macrophage^++^ with intestinal tumor.** A** and **B**. 12 mo TC^–^CD5^–^ ATA B with spleen^++^ generate similar to old aged TC^–^CD5^–^ATA B tumor. TC^+^Tg ATA B CLL/lymphoma are higher NF-kB and TCL1 induced miR21 and miR155 and down miR29. **C** TC^–^ lymphoma/leukemia showed CD11b^++^CD22^++^ and generate Hamp2^++^ (TC^–^ZAP70^–^ are more IL10 and IL-6, and TC^–^ZAP70^+^ are high IL-22R) with iron down, as USF2^+^, TFR^+^, FPN^–^. **D** TC^–^ ATA B lymphoma/leukemia generated high Hamp2 and CD44^++^CD24^low^. Human V2-5 positive papers, as kidney, Breast cancer, Pancreatic cancer, SARS-CoV-2, and SLE. Breast cancer are CD44^++^CD24^lo^. **E** Some ATA B tumor showed together with high myeloid cells (CD11b^+^Gr-1^+^) [13], and V_H_8-12/V_k_19 AGcA generate MZ B with Hamp2^+^ in 12 mo, then old aged generate macrophages^++^ in spleen with intestine/color tumor^++^

We next performed microarray gene expression analysis of tumor to compare TC^–^ versus TC^+^ Tg ATA B cells and ZAP70^**–**^ versus ZAP70^+^ cells in Fig. 4A, B. We also compared gene expression in CD5^–^ vs CD5^+^ ATA B1 cells from 12 mo TC^–^ ZAP70^**–**^ mice in Fig. 5A.

### Increased ZAP70 expression in TC^+^ mice as in TC^–^ mice are similar to human CLL/U-CLL, however, old aged TC^–^ATA B cell tumors increased than middle age TC^+^

Gene expression in ATA B cell tumors compared between old aged TC^–^ZAP70^–^CD5^–^, TC^–^ZAP70^+^CD5^+^, vs middle aged TC^+^ZAP70^–^CD5^+^ mice (Fig. 4A), and between TC^–^ZAP70^–^CD5^–^ vs TC^+^ZAP70^+^CD5^+^ mice (Fig. 4B). Nod1 expression was decreased in old aged TC^–^ tumor cells, and T-bet and CD11c were increased. T-bet^+^CD11c^+^ are consistent with old aged in humans [71, 72]. Tumor from TC^–^ZAP70^–^CD5^–^ mice expressed the highest levels of CD11b and cyclin D2, and TC^+^ (both ZAP70^–^ and ZAP70^+^) ATA B cells lacked CD11b, but TC^+^ZAP70^+^ increased cyclin D2 (Fig. 4B2, B1). Normal B1a cells show higher levels of cyclin D2 than FO B cells (Fig. 2D), and cyclin D2 is expressed in human CLL [73]. Since CB.70 mice have a p16^INK4a^ mutation [74], cyclin 2 (G1) expression can be more increased, and promote moving from G1 to G2/M [75]. Then, both ZAP70^+^ with TC^–^ and TC^+^ are higher in cyclin D1 (G1), cyclin E1 (G1/S), cyclin A2 (G2/M) (Fig. 4A1, B1) (also higher cyclin D3 and cyclin B1 (G2/M); data not shown) by p16^INK4a^ mutation.

ZAP70^+^ tumor under both TC^–^ and TC^+^ genotypes exhibited increased expression of TNFa, CD40, IL4, several Tubb (β-tubulin) genes, cMyc, Fzd2, Ki67, and CD86 (Fig. 4A1, B1). Among the Tubb genes, WT fetal/neonatal B1a are higher in Tubb6 [76], and all B cells are positive for Tubb5 [77]. Tubb5 is high in TC^+^ZAP70^+^ tumor, but Tubb6 is higher in both TC^–^ tumors, as compared to TC^+^ tumor (Fig. 4A1,B3). cMyc was also originally higher in B1a cells (Fig. 2D) and ZAP70^+^ increased as human U-CLL than M-CLL. Fzd2 is β-catenin dependent, and human CLL and ZAP70^+^ U-CLL showed expression of Wnt10a and Wnt6, which generated Fzd2 [78]. Ki67 (NKI67) is a proliferation-related antigen. High Ki-67 index is associated with many cancers, and human CLL ZAP70^+^/U-CLL are also Ki67^hi^ [79]. We also found that CD86 expression was higher than CD80 in ZAP70^+^ tumors (Fig. 4A1 and B1), which was also found in ATAμκTg Rag1KO Thy1KO mice that are ZAP70^++^ (Fig. 2D). As in Figs. 4B1, human U-CLL are most similar to ZAP70^+^ TC^–^ and TC^+^ tumors as increased CD40, IL-4, cMyc, Fzd2, Ki67 (see Fig. S4-1 for human CLL/U-CLL references). Since human CLL with normal p16^INK4a^, human CLL expression of cyclin D1 is limited, and cyclin A2 is not increased, and CD86^+^ cells are only small portion of the CLL [80].

TC^+^ZAP70^+^ also increased as in TC^–^ cells including TC^–^ZAP70^–^ cells (Fig. 4B2). B1a cells showed slightly higher TLR7, TLR9, and TLR4 expression than FO B cells at the 2 mo stage. As intracellular Nod1 is decreased in old aged, TC^–^ promote TLR7 and TLR9 increases (Fig. 4A2), and TC^+^ZAP70^+^ cells also showed increased TLR7 (Fig. 4B2). In contrast, middle aged TC^+^Tg tumor cells are strongly high for TLR4 (Fig. 4A2). Wnt/β-catenin family members are Wnt1,2,3,8,10 with LRP5/6, and non-β-catenin (Wnt/Ca2^+^) family members are Wnt 4,5,6,7,11,16 with ROR1/2. TC^–^ tumors showed dominant expression of Wnt/b-catenin Wnt10b (Fig. 4A2) and also TC^+^ZAP70^+^ tumor showed dominant expression of both Wnt10a and 10b (Fig. 4B2). Previous studies showed that human CLL express Wnt3, 4, 5a, 5b, 6, 7, 10, 11, 14, 16 and up-regulated in CLL are Wnt3,5,6,10,14,16 [81]. Wnt5a/5b are the most important in U-CLL [82] and other human cancers also express Wnt5a, whereas Wnt5a was very low in the mouse ATA B tumors. Rather, Wnt5b and Wnt6 were high in both TC^–^ZAP70^+^ and TC^+^ cells (Fig. 4A2, B2). Wnt10 induces expression of Fzd1 and Fzd5, and Wnt5b induces Fzd4 and Fzd6. ROR1 is commonly expressed in human CLL as a component of the Wnt5a-ROR1-ERK1/2 pathway [83], however, mouse TC^+^ ATA B tumors showed very low ROR1, but instead expressed ROR2. Higher in TC^–^ZAP70^+^ and TC^+^ZAP70^+^ increased Wnt5b–ERK2, also in human U-CLL [54]. Myd88 generated from TLRs and higher in TC^–^ with TLR7, 6 (Fig. 4A2), then, increased Myd88 in TC^+^ZAP70^+^ cells occurred together with TLR7 increase (Fig. 4B2). TC^–^ZAP70^–^CD5^–^ showed increased STAT3, IL-10, IL-6 more than TC^–^ZAP70^+^CD5^+^, and TC^+^ZAP70^+^CD5^+^ cells showed increased these (Fig. 4B2). Thus, middle age TC^+^ZAP70^+^ increased as in some TC^–^ cells (including TC^–^ZAP70^–^CD5^–^) than TC^+^ZAP70^–^. TC^–^ positive cells and increased TC^+^ZAP70^+^CD5^+^ in ATA B tumor are the most similar to human CLL data, and CD44^++^ Ki67^+^ as ZAP70^+^ higher (also TC^–^ ZAP70^–^ positive) are significant in U-CLL (Fig. 4B2, References in Fig. S4-2).

In TC^+^ (ZAP70^–^ and ZAP70^+^) ATA B cells, TLR4, Wnt5b/Wnt6, and Fzd6, Rac2 showed high positive than TC^–^, and Runx2, NF-kB, p300 were also high (Fig. 4A2). Runx2 and p300 are NF-kB generation [84, 85]. Then, TC^+^ showed high APRIL, contrast to TC^–^ showed high BAFF. Tuft1 (tuftelin) is multiple cancers as metastasis, and up-regulating the Rac1/β-catenin pathway [86]. Tuft1 can induce NF-kB [87], and TC^+^ showed high Tuft1 than TC^–^ cells. Although Wnt5a/b from the origin as non-β-catenin, however, Wnt5a/b can be changed to binding to β-catenin [88, 89], thus Wnt/b-catenin-TCF/LEF binding can occur. TC^+^ showed LRP5^+^ (different to TC^–^LRP6^+^) and high LEF1 than TC^–^ (both ZAP70^–^ and ZAP70^+^) (Fig. 4A2, B2).

CD38, CD43, CD44 in B1a cells are originally higher than FO B cells (Fig. S2B), and increased these in old aged TC^–^ tumor than meddle aged TC^+^ cells, as also shown in flow cytometry analysis (Fig. 4A3). CD38 is different from human than mice, since human FO B cells are negative, then GC B cells increased CD38 [90], and human U-CLL showed high CD38^++^ and CD44^++^ [91, 92]. Since high CD44^++^ in human ZAP70^+^ CLL, CD44 is the highest in TC^–^ZAP70^+^ than TC^–^ZAP70^–^, and also TC^+^ZAP70^+^ increased (Fig. 4B2). CD43 showed human CLL positive [93], as found in mouse TC^–^ tumor. TC^+^ showed higher CD21, CD23, CD24, CD27, and CD49d than TC^–^. CD21 and CD23 are original low/negative in PerC B1a cells, and TC^–^ ATA B tumor were similar low/– and increased in TC^+^ ZAP70^–^ cells (but, TC^+^ZAP70^+^ down). Human U-CLL can be CD21^low^ [94], and some CLL also showed low CD23 [95]. CD24 are all normal B cells^+^ and increased CD24 have been known for cell growth/cancer [96], and increased CD24 was found in TC^–^Rag1KOThy1KO mice in mature &middle aged ATA B tumor (Fig. 2D), then, old aged TC^–^ showed decreased (Figs. 4A3 and 5D). CD27 are low/– for all normal B cells compared to CD27^+^ T cells in mice, and TC^+^ cells ( ZAP70^–^ and ZAP70^+^) showed slightly increased CD27. In human CD27 are different from mice, since human CD27 are positive in B cells, and original early human B1 cells are CD27^+^ [97] and CLL CD27^+^ [98], however, U-CLL can become CD27^low/–^ by principal component analysis [99]. CD49d are all B and T cells positive, and increased in TC^+^ Tg cells, in comparison, TC^–^ cells showed down regulated CD49d (Fig. 4A3). Most human CLL are CD49d^+^, however few human CLL can became down regulated CD49d in age [100]. These CD case papers in TC^–^ > TC^+^ and TC^–^ < TC^+^ are also listed in Fig. 5A.

Although ZAP70^+^ increased in TC^+^ cells as in TC^–^ cells similar to old aged human CLL and U-CLL, many showed increased old aged TC^–^ cell tumor than middle aged TC^+^ cells (Fig. 4B3), incruding Hamp2 increased in TC^–^. It is possible that TC^+^  > TC^–^ data could be for TC^–^ decrease in old aged. Thus, next in Fig. 5, 12 mo TC^–^ cells analysis with normal CD5^+^ ATA B mice versus CD5 decreased CD5^–^ ATA B mice with spleen increased mice, compare with original Fig. 4 TC^–^ > TC^+^ and TC^+^  > TC^–^ data. Also, further analyses to check with human old aged CLL related, including CXCR.

### TC^–^CD5 down ATA B cells with spleen^++^ similar to old aged TC^–^ lymphoma/leukemia. TC^+^Tg ATA B tumor are NF-kB1 increased

Figure 5A and B. Middle age 12 mo ATA B TC^–^ATAμκ Tg mice compared between normal CD5^+^ B1a cells versus decreased CD5 (CD5^–^)B1 cells with spleen increase. This TC^–^12 mo analysis checked with original TC^–^ vs TC^+^ ATA B tumor data in Fig. 4. Further, add microarray analysis about β-catenin/CTNNB1, Damp/HMGB1, CXCR (CXCR4, CXCR5) (also CXCR3 in Fig. S2A), DDP4(CD26), AID, CD1d, and CD180 with original Fig. 4A samples, and TC^+^ZAP70^+^ tumor comparison are in Fig. S2. Under 12 mo TC^–^ZAP70^–^CD5^–^ cell, Nod1, IL5R, and CD1d are decreased similar to old aged TC^–^ATA B tumor, and CTLA4 down as both TC^–^ and TC^+^Tg cells (Fig. S2A) and human CD38^++^ as U-CLL are CTLA4 decreased (Fig. S4-3). This TC^–^ZAP70^–^CD5^–^ cell most similar to increased in old aged increased TC^–^ and TC^+^ZAP70^+^  > TC^+^ZAP70^–^ and TC^–^ > TC^+^ ATA B tumor generation (except low Wnt10a), and similar to low TC^–^ cells than TC^+^ cells.

β-catenin/CTNNB1 and HMGB1 are TC^–^ cells high, and TC^–^ ZAP70^+^ are more higher, and TC^+^ ZAP70^+^ are also increased (Fig. S2A). Damp, as damage-associated molecular patters (tumor promoting, and anti-tumor effects), present HAGB1 (high mobility group box 1) [101], and Damp/HAGB1 generate TLR pathway (TLR2,4,7,9), also generate CXCL12 with CXCR4 [102]. High β-catenin CTNNB1 and HMGB1 in TC^–^ and TC^+^ZAP70^+^ cells are in human CLL positive and high HMGB1 in CLL plasma [103].

About detail of CXCR in Fig. S2A. CXCR4 is TC^**–**^ high and TC^+^ low, as TC^+^ZAP70^+^ also low. Then, decreased CXCR5 in TC^–^, and TC^+^ZAP70^–^ with continuously high CXCR5 as original 2 mo B1a cells, however, decreased CXCR5 in TC^+^ZAP70^+^. CXCR3 and CCR7 were also higher in TC^–^ than TC^+^ZAP70^–^, and TC^+^ZAP70^+^ with decreased CXCR5 showed slightly increased CXCR3 but CCR7 was not increased (Fig. S2A). Thus, TC^–^ATA B tumor showed CXCR4^+^, CXCR3^+^, CCR7^+^ and low CXCR5. In human CLL, CXCR4 higher than CXCR5 [70] different from mouse TC^+^Tg, and CLL also showed CXCR3^+^ [104] and CCR7^+^ in CLL. DPP4^+^ (dipeptidyl peptidase-4)(CD26) are majority of all B cells^+^, and DPP4 also increase CXCR4 [105]. DPP4 are higher in old aged TC^–^ with CXCR4^hi^, and low in middle aged TC^+^ cells (both ZAP70^–^ and ZAP70^+^) as CXCR4 low. Human CLL are high DPP4, and with high DPP4 in serum with U-CLL [106, 107]. AID (activation-induced cytidine deaminase) original in fetal liver [108], and autoreactive B cells in immature B cells (T1/T2 B cells) in B-2 cells are AID^+^, then down for mature FO B, and B1a are also originally low AID (Fig. S2A), then, TLR9 induces AID [109]. In human, U-CLL showed high AID than mutated M-CLL [110], and mouse TC^–^ ATA B tumor in old aged, also showed high AID, not TC^**+**^ cells (both ZAP70^–^ and ZAP70^+^) (Fig. 5A, Fig. S2A). CD1d and CD180 are decreased in old aged TC^–^ATA tumor, compaired to middle aged TC^+^ (both ZAP70^–^ and ZAP70^+^) (Fig. 5A, Fig. S2A). CD180 is originally increased from immature to mature FO B and MZ B cells, also B1a cells CD180^+^. Then, TLR7,9 signaling pathway significantly downregulate CD180 [111] and mouse old aged TC^–^ ATA B tumor showed the lost CD180. In human old aged CLL, survival mature M-CLL are CD180^+^, contrast to U-CLL are CD180^–^ [112], and aged memory B cells became CD180^–^. Conclusion, TC^–^ > TC^+^ and also down TC^–^ in old age were most similar to 12 mo spleen infection in TC^–^CD5^–^ cells, and these are most similar to old aged human CLL/U-CLL (TC^–^ > TC^+^ samples for human CLL/U-CLL references in Fig. S4-2).

TC^+^  > TC^–^ tumor lists found to be mostly down in 12 mo TC^–^CD5^–^ cells than normal TC^–^CD5^+^B1a. Thus, most TC^+^ tumor appeared to be not increased by tumor than TC^–^. However, 12 mo TC^–^CD5^–^ increased high NF-kB1 and also increased in Runx2 and p300 as similar to TC^+^  > TC^–^. Thus, clearly, TC^+^ cells are more increased NF-kB1 and NF-kB related Runx2 and p300 [84, 85] than TC^–^. In Fig. 5B showed that NF-kB is also higher in TC^**+**^ZAP70^+^ than TC^–^. TCL1 leads AKT [113] and mostly AKT2-NF-kB [114]. AKT2 are similar in TC^–^ to TC^+^ZAP70^–^, however, TC^+^ZAP70^+^ are increased. Human TCL1 showed higher miR21 and miR155, and lower miR29, and in ATA B cells, these miR showed TC^+^  > TC^–^ (Fig. 5B) similar to human TCL1^+^ CLL [115, 116]. However, down miR15a and miR16 to mediate Bcl2 increase as found in human CLL [117], and similar by down in TC^–^ than TC^+^ (Fig. 5B). Low CD21, CD23, CD24, CD27 and CD49d in TC^–^ ATA B tumor mice are also low in 12 mo TC^–^CD5^–^ spl^++^ cells, and these low data can be found in some in U-CLL (CD21, CD27) [94, 99] or low generated (CD23, CD49d) can occur in few CLL [95, 100]. Since normal CD5^+^ATA B cell and TC^+^Tg tumor with CD5^+^ samples are most human TC^+^ CD5^+^CLL positive, these in human CLL references also showed in Fig. S4-3. Hamp2^++^, CD11b^++^ and down CD24 in old aged TC^–^ ATA B tumor are similar to 12 mo TC^–^CD5^–^ cells, but these are not in human CLL.

### Increased CD11b^++^CD22^++^ and Hamp2^++^

Figure 5C. Originally, B1a cells are CD5^+^ which bind to SHP-1 as Lyn–CD22–SHP-1. CD22 is from BCR [45], then binding CD5–SHP1 or CD11b–CD22 inhibits BCR signaling [46]. When loss as CD5^–^ and CD11b^–^ in spleen B1 [48], middle aged become CD11b^+^ such as by CpG bacteria or CpG^+^IL-10^+^ increase, and in old aged, TC^–^ ATA B showed increased CD11b^++^, then increased CD22, and TC^–^ZAP70^–^CD5^–^ are more higher CD11b^++^CD22^++^ (Fig. 5C). CD22^++^ found in HCL (hairy cell leukemia) [118], however, human CD5^+^CD11b^–^CLL is CD22^lo^ not require CD22^++^, thus different [118]. TC^–^CD11b^++^CD22^++^ is allowing the migration and maintaining autoreactive B cell tolerance, and SLE (Systemic lupus erythematosus) found CD11b^++^CD22^hi^ (Fig. 5D) also autoreactive [46]. α-Thy-1 ATA B cells with CD11b^++^ can Thy-1 moving to endothelial to control virus infection as by CD11b^+^Gr-1^+^ endothelial tissues (33.34).

Hamp2 also increased in TC^–^ ATA B cells in old aged. In hepcidin, mouse ATA B cell tumor did not show Hamp1 but Hamp2^++^. Hepcidin is important for systemic iron homeostasis, and hepcidin generations are the most CD11b^+^Gr-1^+^ macrophage and monocytes, and TLR induced hepcidin in T cells and B cells [42]. Hepcidin control iron is most in liver, however, mouse Hamp2 is in both liver and pancreas with antibacterial and antivirus active types, and B cells including B1 cells can move from spleen to pancreas with high Hamp2 [119], and Hamp2 is not change erythropoiesis different from human hepcidin [120]. In hepcidin, IL-10, IL-6, and IL-22 generate Stat3, then regulate for the induction of hepcidin [121, 122]. In mice, fetal and neonate showed IL-10 and IL-6 [123, 124]. IL-10 is original B1a cell generation, however, IL-6 did not require for B1a, rather, IL-6^–^ showed increased B1a cells [125]. Different from Arid3a which is important for neonate mature B1a generation, Arid5a is more adult immature B cell stage and increased IL-6 for B cells [12, 126]. IL-6 reacts with Th2 and Th17, and mostly increased in Th17 [127]. Since in old age, T-bet and CD11c increased [2], T cells changed Th1 and Th17 increase, and Th2 decreased with B cell responsive as IL-4R down (Fig. 5C). High IL-6 in old aged ATA B cell tumor in TC^–^ZAP70^–^CD5^–^ cells are the Arid5a generation with IL-6^+^ [126] (Fig. 5C) and also IL-10^++^ (Fig. 4A2). Iron regulation require USF2-hepcidin to generate iron [128, 129]. Strongly USF2 high in TC^–^ cells in ATA B cells (Fig. 5C). In TC^–^ZAP70^–^ were high IL-10 and IL-6 than TC^–^ZAP70^+^ cells, and higher IL-22 in TC^–^ZAP70^+^ in USF2 and IL-22R (Fig. 5C). In Th17 cell, IL-17 and IL-22 generation [130] and TC^–^ATA B tumor showed highest IL-22 to receptor IL-22R in the highest TC^–^ZAP70^+^ cells as Hamp2 highest generation (Fig. 4A2). IL-22 also generated by innate lymphoid cells ILC3 [131]. Cleary, Hamp2^++^ TC^–^ ATA B tumor cells decreased iron stage, by TFR1^+^ high (as origin iron) and negative ferroportion FPN (SLC40A1) as iron reduction with TFR1^+^FPN^–^ [132]. Thus, old aged TC^–^ATA B tumor increased Hamp2^++^ and decreased iron (Fig. 5C).

### V2-5 human cancers with hepcidin^+^ or iron^+^ as mouse V8-12 ATA B1 cell Hamp2^+^ and AGcA NZ B-macrophage-intestine tumor. V2-5 breast cancer is CD44^++^CD24^lo^ as old aged ATA B cells

Figure 5D and E. Mouse V8-12 is similar to human V2-5, and HIV-1 virus showed V2-5 and V1-69 generation, and V2-5^+^ in Ankylosing spondylitis, Alzheimer’s disease, and Multiple sclerosis [61–64, 66]. And, human normal kidney present Thy-1^+^ with V1-69 and V2-5 positive, and several cancers with including V2-5.

- Kidney: V3-23, V1-69, V2-5 [133]
- Breast cancer: V1-69, V2-5, V3-49, V3-64, V4-59, V5-51 [134]
- Pancreatic cancer: high mutatedV3-66, V3-9, V3-38, and low V1-2, V2-5 [135]
- SARS-CoV-2(Covid-19): V2-5 or V1-6, V3-53, V2-5 [136, 137]
- SLE (Systemic lupus erythematosus): J6 = V2-5, V3-35, V3-33, V7-4, V3-36, V2-26 [138]
- In the SLE group, common usage frequencies of the V gene and J gene, and the proportion of IGHJ6 are higher in SLE group

Mouse V8-12 ATA B cells (anti-thymocyte/Thy-1 and polyspecific) are not only CLL, but old aged ATA B cells related to cancer Spl^++^, mLN^++^, LN^+^, Liver^++/+^.

In human, although T cells are completely absent Thy-1, Thy-1 are present. Thy-1 can move to endothelial stage for tumor suppressor, such as in virus infection, and this Thy-1 move to endothelial cells with dominantly from CD11b in CD11b^+^Gr-1^+^presenting monocytes /macrophase [33, 34] in human. In both human and mice, Thy-1 is in brain neuron together with CD11b^+^ macrophage [139, 140], thus Thy-1 is important. In human, Thy-1^+^ is also present in kidney in normal in epithelial cells (ECs) [141], also, urine as a liquid by-product of metabolism generates hepcidin^+^ [142] (Fig. 5D). As show in Fig. 5D, several cancers also showed Thy-1^+^ and hepcidin^+^. In breast cancer, Thy-1^+^ are present in epithelium ECs together with some MSC (mesencymal stem cells) and in endothelium cells (ECs), and hepcidin^+^ and iron deficiency [143]. In SARS-CoV-2, epithelium ECs and MSC with Thy-1^+^, and hepcidin^+^ with iron decreased [144]. In SLE, most MSC are CD90 (Thy-1), CD105, CD73 positive, and hepcidin^+^ and iron decreased [145]. Human pancreas is different from mouse in Hamp2^+^ in pacreas [119], and in human pancreatic cancer, fibroblasts and endothelium ECs present Thy-1, however, hepcidin low and iron overload [146], with high macrophage and neutrophil interaction in pancreatic tumor microevironment [147], not normal increases hepcidin. Thus, pancreatic cancer is the different paper to generate high macrophage with low hepcidin and iron overload.

CD44 is homing receptor and original pB1a are higher than FO B cells (Fig. S2B), and more increased in old aged ATA B tumor. CD24 (30F1) is a glycophosphatidylinositol (GP1)-anchored protein, with all normal B cells express. CD24 overexpressed in tumor and human CLL [96], and mature & middle ATA B tumor under Rag1KOThy1KO mice showed also higher CD24, then, old aged ATA B tumor showed down CD24 (Fig. 5D). CD44^hi^CD24^–/low^ are well known for human breast cancer [148], originally generated by EMT (epithelial-mesenchymal-transiton) [149]. EMT plays a key role in the regulation of cell motility and invation. In pancreatic cancer, CD44^hi^CD24^hi^, but when non-B cell EMT presented CD44^hi^CD24^–^ [150]. DAMP/HMGB1 can present EMT [151], and AID also increased EMT [152]. Since old aged ATA B tumor showed DAMP/HMGB1^+^ and AID^+^, it is possible that EMP increased with changed to CD24^low^ with CD44^++^. Ki67 is increased from HMGB1 [153], and TC^–^ZAP70^+^ showed both highest Ki67 and HMGB1, then lowest CD24 in TC^–^ZAP70^+^ than TC^–^ZAP70^–^ (Fig. 4A3). However, also, p16^INK4A^ reduction increase EMT [154], and C.B17 mice are p16^INK4A^ mutation and human breast cancer are often p16^INK4A^ mutation (49%) [155], thus, ATA B CD24^low^ with EMT may be the more generated incruding by p16^INK4A^ reduction in old age.

Similar to old aged mouse ATA B tumor, human breast cancer showed CXNNB1^+^, HMGB1^+^, Ki67^+^, CXCR4^+^ and serum DPP4^+^. Pancreatic cancer showed CTNNB1^+^mutation, HMGB1^+^, Ki67^+^, CXCR4^+^, DPP4^+^. SARS-CoV-2 showed, CTNNB1^hi^, and serum HMGB1^hi^ and dominant T cells showed Ki67^+^, and CXCR4^+^, DPP4^++^. In SLE, CTNNB1 low but urokinase-type plasminogen activator made CTNNB1^+^, and serum HMGB1^+^, and Ki67^+^, CXCR4^+^, DPP4^+^. Then, SLE originally known by presented CD5^+^ B cells, then, CD5 decreased B cells can occurred, also CD1d downregulated. As found in mouse old aged ATA B tumor, SLE are known the autoimmune disease and generated CD5^–^, CD1d^–^, CD22^hi^, CD11b^++^, AID^+^, CD180^–^, CD21^–^, CD27^–^ (Fig. 5D), also T-bet^hi^ CD11c^hi^ and TCR7^+^ TCR9^+^. All reference in Fig. 5D listed in Fig. S5: Brest cancer, Pancreatic cancer, SARS-CoV-2, and SLE.

Figure 5E. B cells can cross talk fibroblast and became to epithelial cells [156], and B-1 B1 B cells and B-2 MZ B cells interacts macrophage and dendritic cells [157, 158], and the spleen MZ B cells clearly contains macrophage receptor. As human CLL showed some increased myeloid cells, old aged mouse ATA B tumor showed increased CD11b^+^Gr-1^**+**^ myeloid cells/macrophage with 12% (Fig. 5E) [13]. V8-12 μTg cells showed increased in MZ B with V_k_19-17 as AGcA, and generated V8-12/V_k_19-17μκTg mice strongly AGcA MZ B cell generation at first 3 wk as early B-2 stage [54] (Fig. S3), and this AGcA MZ B cells showed increased Hamp2 at 12 mo, as ATA B1 cells Hamp2^+^, however V11-2 aPtC B1a cells are not increased Hamp2 (Fig. 5E). In old aged, originally AGcA MZ B cells can become tumor 7%, but macrophage with CD11b^+^Gr-1^+^ are most strongly increased in spleen by MZ B cells as spleen^++^ (56%), and high intestine/colon tumor generated (Fig. 5E, and pictures in Fig. S3), also resulting in a upper respiratory swollen neck (Fig. S3). Human HIV-2 virus are V2-5 positive [61] and accumulated macrophage and hepcidin increases with iron decrease, thus maintenance of viral set-point [159, 160]. Ankylosing spondylitis (AS) with most V_H_2 (dominant V2-5) [62] are the neutrophils progression, and Alzheimer’s disease in brain (AD) found in V2-5 are with macrophage polarization subsets, most in bacteria [161]. Both AS and AD showed hepcidin^+^ plays with an important role [162, 163]. Multiple sclerosis (MS), also V_H_2 (dominant V2-5) positive [66], as a brain-gut axis and blood–brain, with important macrophage [164], however, with low hepcidin and crucial role of iron in serum [165]. Clearly, increased iron in brain in MS in age [163], and MS is intestinal barrier occur with iron [166, 167]. Aging spleen macrophage can increase tumor-derived factor with iron^+^ in mice [168] and macrophage-iron^+^ in old/damaged generate red blood cells, generate brain functions (brain-spleen axis) and generated colon (spleen-gut axis), then gut-brain-spleen axis [166]. These iron^+^ in tumor generation in human is TFR1^hi^ and FPN^+^, not FPN^lo^ [169]. Human V2-5^+^ cancer with macrophage/neutrophils generate mostly high hepcidin with iron deficiency, however, pancreatic cancer and multiple sclerosis (MS) are macrophage generate with high iron with low hepcidin, similar to mouse V8-12^+^ AGcA MZ B increased macrophage to generate intestinal/colon tumor. Clearly, human V2-5 homologous to mouse V8-12. Mouse old age ATA B cells showed CD11b^++^ with control Thy-1 and directly Hamp2^++^ and some increased macrophage, and MZ B showed strongly macrophage increased in spleen with tumor-derived factor.

### IgVH in TC^+^Tg lymphoma/leukemia

**Fig. 6:**
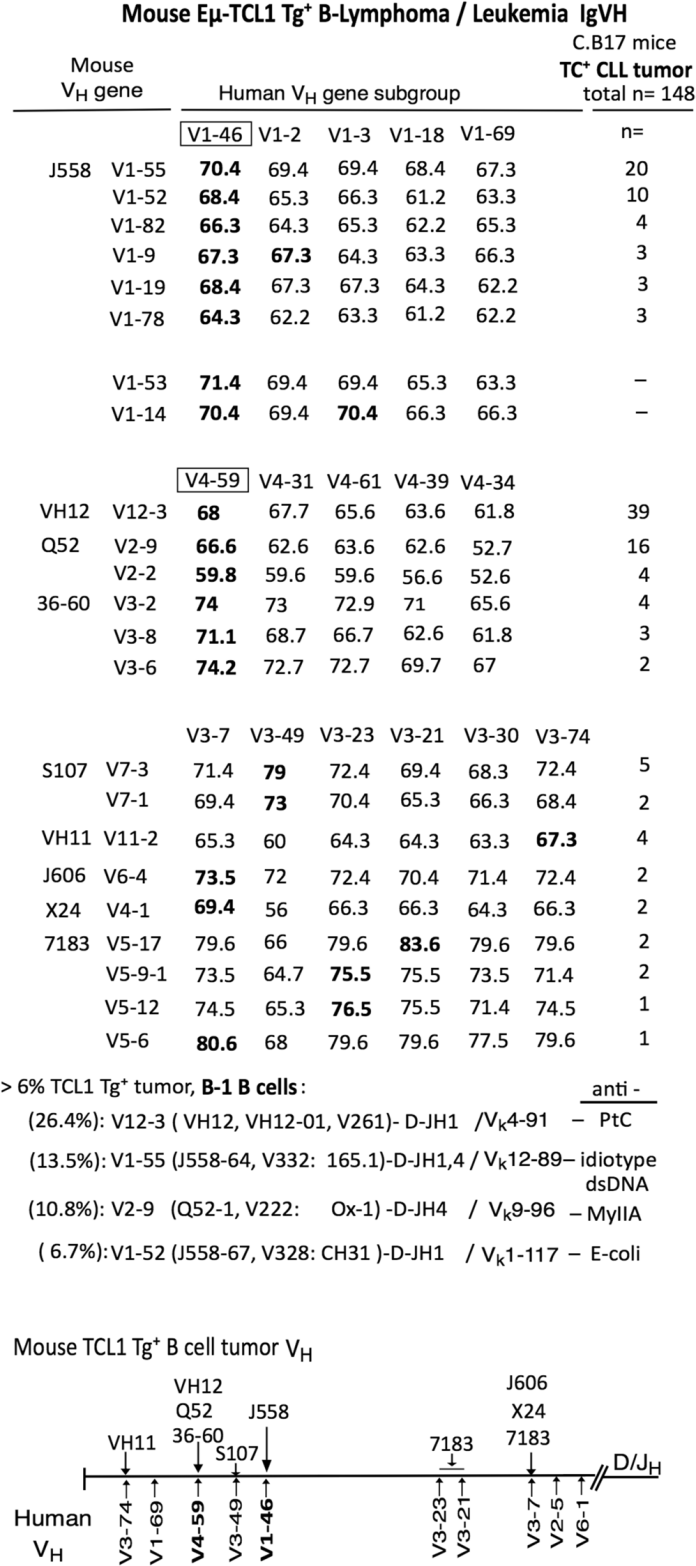
Mouse TC^+^Tg B-lymphoma/leukemia IgVH list. TC^+^Tg C.B17 mice generated CLL tumors were previously published [24], then, found mouse V1 generated tumor is slightly similar with human V1-46, and mouse V12, V2, and V3 generated tumor are similar with human V4-59. Mouse V7, V11, V6, V4, V5 generated tumors are each different in human V3. Higher TC^+^Tg tumors (V1-55, V1-52, V12-3, V2-9) are with mouse non-mutated B-1 B cell origin

TC^+^Tg ATA B cells are different from TC^–^ old aged tumor. We previously published TC^+^Tg generated tumor and found most VHs are as B-1 cell CLL tumor [24] (Fig. 6). In this TC^+^Tg C.B17 mice, generation of ATA B cells were low (than SM/J and NZB mice), rather, showed several increased IgV_H_. Dominantly high in J558 (V1-55, V1-52), V_H_12 anti-PtC (V12-3), Q52 anti-MyIIA (V2-9). In J558 V1 cells, TC^+^Tg generation (V1-55, V1-52, V1-82, V1-9, V1-19, V1-78), and in normal C57BL/6 B1a cells V1-55 and V1-53 are high beginning at 2-3wk [170], and V1-14 as anti-Sm for B1a cell. All these V1 showed slightly increased level in human V1-46. Human CLL is low V1-46, however, ratavirus-specific B cells showed high V1-46 [171], and, when repertoire of rotavirus generation, young children showed highest V1-46 [172].

V_H_12 (V12-3), Q52 (V2-9, V2-2), 36–60 (V3-2, V3-8, V3-6) showed slightly reacted to human V4-59 (lower V6 than V4-59). In both cord blood and adult blood in human, V1-69 and V4-59 are the higher list, and in cord blood with depth analysis, V4-59 > V4-43 > V2-5 [173]. Also, in older subjects, V4-59 is also positive. In human CLL, V4-59 is the medial level with mutated or some non-mutated [174]. V4-59 also occurred in HCL (hepatitis C virus) [175], and MCL (mantle cell lymphoma) showed V3-21(mutant) and V4-59 (not/low mutate) [176].

S107 (V7-3, V7-1) are positive with human V3-49, V_H_11 (V11-2) is positive with V3-74, J606 (V6-4) and X24 (V4-1) are positive with V3-7, and 7183 (V5-17, V5-9, V5-12, V5-6) showed different V3 positive. In human CLL, V3-7 and V3-23 are positive (V3-7 are mostly mutant, and V3-23 are low mutant or mutant), and V3-49, V3-21, V3-74 are low in CLL [174]. Mouse aPtC V11-2 are well known for mouse B1a cells, and human V3-74 showed high in celiac disease (CeD)-specific antibodies in gut (gastrointestinal tract), and human aPC showed V3-74 [177]. Since mouse V1-55 and V1-52 (similar to human V1-46), and V12-3 and V2-9 (similar to human V4-59) were higher in TC^+^Tg cells, we next checked the region for higher these V_H_ cells in TC^+^ Tg B-1 tumor cells.

### High increased B-1 VH cells generated in TC^+^Tg are not only related to fetal/neonatal generation

**Fig. 7:**
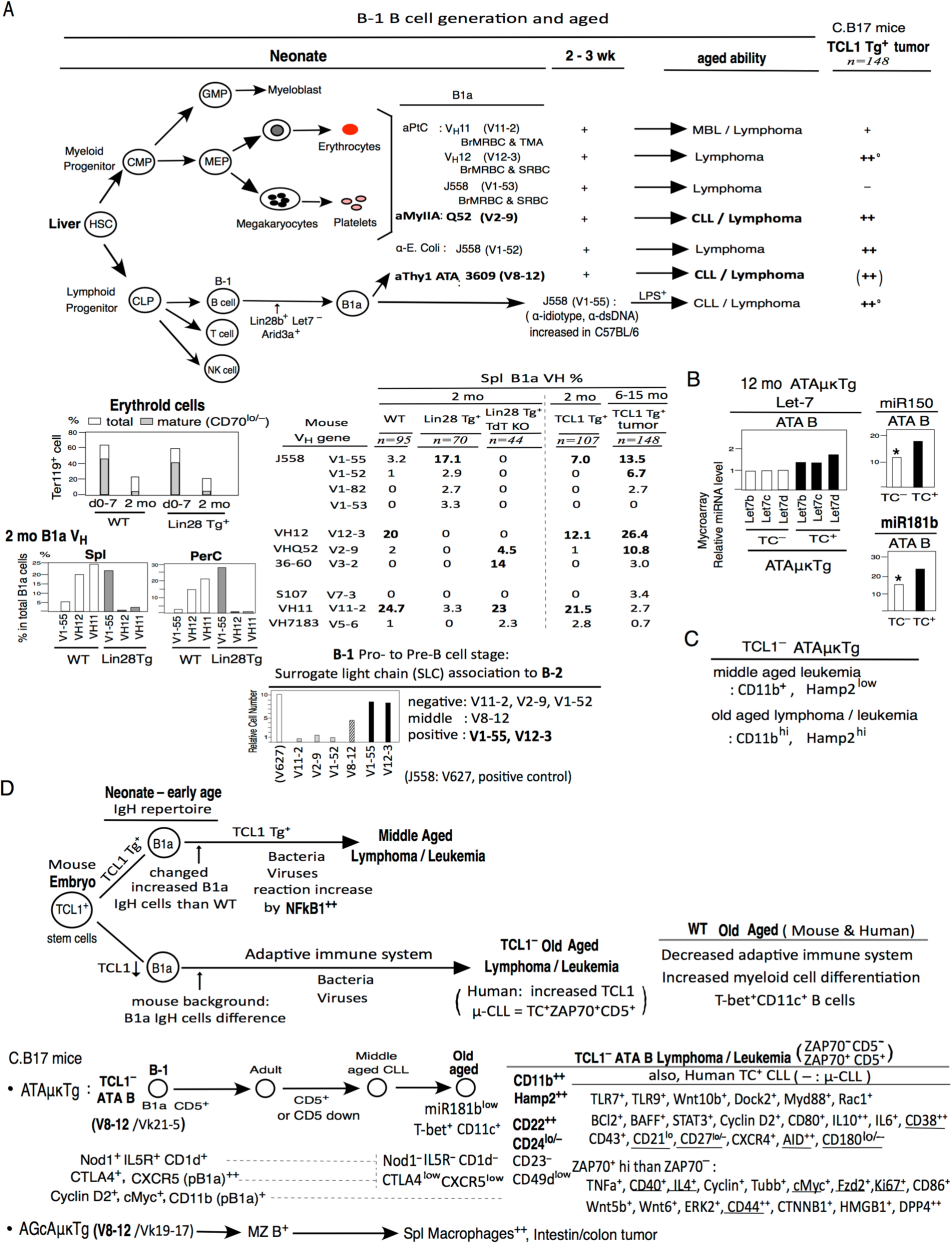
TC^+^Tg B1 B cells in neonate to early age are changed than TC^–^. Old aged TC^–^ATA B cells similar to human CLL/U-CLL and further some different. **A** Summary. In B-1 B cell generations, aged ability for lymphoma or CLL/lymphoma [13, 25, 178, 180], and V1-55 in C57BL/6 mice is high at 2-3 wk, than 1 wk [170]. Down: 2 mo Lin28 Tg^+^ changed are high V1-55 and decreased V1-55 with negative TdT^KO^ Lin28 Tg^+^ persentage, as TdT requaded for B-2 from Pro-B (TdT^+^) move to Pre-B. When V1-55 and V12-3 B-1 Pro-B to Pre-B cell stage with heavy chain with surrogate light chain (SLC) association to B-2 analysis. Thus, increased in TC^+^Tg. **B** 12 mo microarray relative miRNA level Let-7 showed slightly higher in TC^+^Tg ATA B cells than TC^–^, and TC^–^ ATA B are low miR150 and miR181b. **C** High increased CD11b and Hamp2 in old age TC^–^ ATAμκTg mice. **D** Concludion of difference of TC^+^Tg and TC^–^ mice to generated lymphoma/leukemia, and in C.B17 mice TC^–^ATAμκTg ATA B from neonate, middle age, and old aged. Old age ATAB lymphoma/leukemia generation with most similar to human TC^+^CLL/U–CLL and different to CLL with CD11b^++^Hamp2^++^, CD22^++^, CD24^lo/–^. CD23^–^CD49d^low^ are the some generated cancers. AGcA generate MZ B and changed to increased macrophages^++^ with intestine/colon tumor in old age

As shown in Fig. 7A, neonate liver HSC (hepatic stellate cells) generate myeloid progenitor (CMP) and lymphoid progenitor (CLP). Innate immune system in CMP generate myeloblast (GMP) and erythrocyte and megakaryocytes-platelets generation (MEP). In CLP, B-1 cells generated under Lin28b^+^Let7^–^ and Arid3a^+^ allowed generation of CD5^+^ B1a cells. Several B1a binds to erythrocyte under aPtC (phosphatidyleholine): V_H_11 (V11-2), V_H_12 (V12-3), J558 (V1-53), and aMyIIA (non-muscle myosin IIA) Q52 (V2-9) also can bind to erythrocytes and platelets. These B1a binding to erythrold cells are high in neonate than adult stage [43] (Fig. 7A). In TC^–^ aged, aPtC (V11-2, V12-3, V1-53) and aMyIIA (V2-9) showed lymphoma [25, 178], and in old aged V11-2 μTg mice (V_H_11t) generated MBC (monoclonal B cell lymphocytosis) in PBL [13], and aMyIIAμκTg (as ON25) generated aMyIIA B CLL/lymphoma [25] (and Fig. S1C). Since human V1-67 and other IgH in U-CLL can bind to MyIIA [179], mouse neonate TC^–^aMyIIA B cell became similar to human TC^+^U-CLL. J558 (V1-52) reacted for E-coli from early B1a cells, also generation of lymphoma. J558 (V1-55) is not found in earliest neonate stage, then, at 2–3 wk high increased in C57BL/6 mice [170], and when LPS^+^ generation, strongly CLL/lymphoma generation occur by V1-55 in age in C.B20 mice [180]. In TC^+^ Tg in C.B17 mice, highest V12-3 and V1-55, and higher V2-9 and V1-52 in CLL [24].

Erythrold cells are lower in 2 mo than neonate stage [43] (Fig. 7A). Originally Lin28b^+^ in neonate generated B1a V_H_11 and V_H_12 are continuously incrused in 2 mo Lin28b^–^ Spl and PerC. In contrast, when Lin28b^+^ Tg generate, V1-55 became highest in 2 mo adult, contrast downregulate V_H_12 and V_H_11 [43] (Fig. 7A). In adult BM, pro-B to pre-B cell generation requires TdT (Terminal deoxynucleatidyl transferanse) for N-necleotides to the V.D. and Jexon of the BCR, in contrast, N-region can not require in fetal and neonate [181]. TdT decrease as TdT KO mice together with Lin 28b^+^ Tg mice showed negative in V1-55, contrast, increased in several earliest generated B1 related cells such as V_H_11 and Q52 (V2-9), although not increased in V12-3. When B-1 pro- to pre-B cell stage by binding to surrogate light chain (SLC) associated in B-2 stage, clearly that V11-2, V2-9, V1-52 are negative, and middle by V8-12, then positive by V1-55 and V12-3, as previously identified [24]. Thus, B-1 V12-3 and V1-55 can continue to generate to B-2 immature B cells not only early B-1 stage, then, TC^+^Tg aloud increased. Anti-PtC V_H_11 and V_H_12 are different. Both react with BrMRBC (bromelain-treated mouse erythrocyte), however, V_H_11 as a stage of TMA(trimethylammonum) present, and anti-TMA response reflects the V_H_11 usage in original mice [182], in contrast, V_H_12 showed high reaction with SRBC(sheep red blood cells) as sheep erythorocytes with continuously increase [182]. Since TC^+^Tg mice continuous generate TC^+^, more generated in V12-3 and V1-55 in age, but, early strongly neonate stage V2-9 (aMyIIA) and V1-52 can also increased in age (Fig. 7A).

In neonate ATA B cells, TC^+^Tg ATAμκTg ATA B mice showed continuously slightly higher Let-7 as adult Lin28^–^Let7^+^ than TC^–^, thus, TC^+^Tg changed. TC^–^ATA B cells showed lower miR150 and miR181b than TC^+^Tg (Fig. 7B). miR150 and miR181b are originally low in neonate B1 cells since Lin28^**+**^ downregulate miR150 and miR181 [183, 184], and well known that original miR150^–^ makes in human B1a cells [185], and human U-CLL are miR150^low^ [186]. In adult miR181 are positive, then, TC^+^ human old aged, miR181 can be decreased, and showed miR181b^low^ is making progression and cell death resistance with human cancer, and miR181 down regulation showed increase TCL1 in human [187]. Thus, old aged mouse TC^–^ ATA B tumor with down miR181b similar to human CLL progression [188], without increased TCL1 in mice. TC^–^ ATAμκTg tumor showed increased CD11b and Hamp2 in middle aged CLL, and further increased in old aged, different from TC^+^Tg. (Fig. 7C).

### Old aged TC^–^ ATA B cell lymphoma/leukemia with old aged human CLL/U-CLL, and increased Hamp2 as in human cancer hepcidin^+^

Summarized in Fig. 7D. TCL1 are positive in embryo, originally. Then, in mice, TCL1 (as TCL1A) down regulated at 18 day in fetal liver as TC^–^ [16], thus, TC^+^Tg generation showed continuously TC^+^ fetal/neonate and aged. Originally generated B1 cells in TC^+^Tg are sertein IgH cells increased in CLL Lynphoma/Leukemia at middle aged with increased NF-kB in reaction to bacteria or viruses generated. Middle age TC^+^Tg mice tumors are not all similar to old aged. In TC^–^B1a cells at neonate-early age stage, mature IgH cell levels are different from mouse background, then, certain original TC^–^B1a cells VHs can generate in old age lymphoma/leukemia. In old aged, both mouse and human generate more T-bet^+^CD11c^+^ as different T cells, and increased myeloid cell differentiation [189]. Then, increased TCL1 in human CD5^+^B cells can occur with sensitive bacteria increase generating CLL. Since CD5^+^ cells leads to stop of BCR signaling, ZAP70^+^ further required for immediate strongly in human TC^+^ZAP70^+^CD5^+^ U-CLL for more aggressive with a significantly short time than M-CLL. In mice, increased ZAP70 can several increase than ZAP70^–^, but CD5 down with high CD11b^++^CD22^++^ in TC^–^ZAP70^–^CD5^–^ can also occurred lymphoma/leukemia generation in old age, and high Hamp2^++^.

In ATAμκTg mice in neonate and early aged, B1a cells increased Nod1^+^, IL5R^+^, CD1d^+^, CTLA4^+^, CXCR5^+^ (in pB1a^hi^), cyclin D2^+^, cMyC^+^, and CD11b^+^ in pB1a (not sB1a), as also normal B1a cells. Since C.B17 mice has p16^INK4a^ mutation, further high cyclin D2. In old aged ATA B cells, decreased originally Nod1, IL-5R, CD1d, CTLA4, CXCR5, also CD49d in B1a cells. In contrast, cyclin D2, cMyc, also CD38, CD44, CD43, STAT3, BAFF, were more increased in old aged and CD11b increased. And, high T-bet^+^CD11c^+^ and miR181b^low^ in old aged are similar to human old aged CLL. In old aged TC^–^ATA B mice, ZAP70^–^CD5^–^ and ZAP70^+^CD5^+^(also CD5^–^), are generated with increased cells or down cells similar to human CLL/U-CLL (Fig. 7D). Different from human CLL, ATA B cells showed often CD5 down by bacteria in aged, then with increased CD11b^++^CD22^++^ by highest in old age, and the Hamp2^++^ with decreased iron. Decreased CD24 in mouse old aged ATA B tumor cells by including from p16^INK4a^ mutated. Human several cancers showed hepcidin^+^ with decreased iron, and hepcidin^+^ increased by CD11b^+^Gr-1^+^ macrophages/neutrophils. Mouse old aged V_H_8-12/V_k_21-5 ATA B cells generated directly high Hamp2 and iron decrease in B1 cells. Different to ATA B1 cells, mouse V_H_8-12/V_k_19-17 AGcA generate MZ B cells increased high macrophage, then, intestine/colon tumor generation.

## Conclusion

Neonate V_H_8-12/V_k_21-5 CD5^+^ATA B cells generated old aged leukemia/lymphoma without TCL1. ATA B cells can decrease CD5 in middle age and increase CD11b^++^CD22^++^ in ZAP70^–^CD5^–^, generating old age leukemia/lymphoma with autoimmune disease and control Thy-1, also ZAP70^+^CD5^+^ with CD11b^+^CD22^+^. This mouse TC^–^ATA B cell tumors in old age most similar to human old age TC^+^ CLL/U-CLL, and middle aged TC^+^Tg ATA B mouse tumors are not similar to old aged in TC^–^ mice, but increased ZAP70^+^ made several increased data as in old aged TC^–^. Certain original generated in mouse B1 cell genes decrease or increase in old age. Then, ATA B cells are hepcidin-related Hamp2 increased with iron^low^ as similar to human cancer hepcidin^+^ iron^low^. Mouse V8-12 similar to human V2-5, and V2-5 related to several cancers with hepcidin^+^iron^low^ generated macrophage/neutrophils, and multiple sclerosis are hepcidin^–^iron^+^ generated from macrophage including brain-gut/colon axis. Mouse V_H_8-12/V_k_19-17 generate AGcA MZ B cells increased macrophage with intestine/colon axis tumor with old age. Conclusion, mouse with V8-12 in old aged, B-1 cells generated CLL/cancer and B-1 cells directly with Hamp2^++^ Iron^low^, contrast, B-2 MZ B cells increased macrophage in spleen with intestinal/colon tumor. Since V_H_8-12/V_k_2-5 B1cells can also interacts macrophages, mouse V8-12 similar to human V2-5 generated macrophage/neutrophil with hepcidin^+^iron^lo^ or hepsicin^–^ iron^+^.

## Materials and methods

C.B17 mice is the BALB/c background with changed as IgM^b^. Originally generated anti-thymocyte/Thy-1 (ATA) B cells (IgM^a^) in SM/J mice [10], and generating ATAμκTg and ATAμTg under C.B17 mice with ATA B lymphoma/leukemia generation in old age [10, 11, 24]. Thy-1KO or Rag1KO under ATAμκTg were generated [55], and ATAμTg generated MZ B cells as AGcA B cells and AGcAμκTg generated dominant MZ B cells were published [54], and continuously analyzed for AGcAμkTg mice in old age, with high macrophage in spleen and generated in intestinal tumor. Eμ-TCL1 transgenic mice (TC^+^Tg) in C.B17 mice, also with ATAμκTg or ATAμTg, and showed generated lymphoma/leukemia [24]. In this paper, microarray gene-expression analysis of ATA B lymphoma/leukemia by old age TC^–^ versus middle age TC^+^Tg in ATAμκTg tumor and 12 mo TC^–^CD5 down ATAμκTg comparison.

### Microarray gene-expression analysis

*mRNA expression analysis* using Agilent Technologies whole genome arrays**.** Total RNA, isolated as described previously [190], was used for production of fluorescent-labeled probe and then hybridized to the array. RNA purity and integrity were evaluated using the 2100 Bioanalyzer (Agilent Technologies) and NanoDrop 1000 (Thermo Fisher Scienti c) before probe generation. Experimental samples were labeled with either Cy3 or Cy5, and a common reference RNA, a pool of total RNAs from several mouse organs generated in the laboratory, was also fluorescent labeled. Each sample was done twice with dye-swap as technical replicates. Hybridized slides were scanned on an Agilent Technologies scanner, and fluorescent intensities of hybridization signals were extracted using Agilent Technologies Feature Extraction software. Statistical analysis was performed in the Fox Chase Biostatistics Facility.

*miRNA expression analysis*. Total RNA was isolated using TRIzol reagent (Invitrogen) and washed with 80% ethanol to better retain small RNAs. The RNA quality and integrity were evaluated using the 2100 Bioanalyzer and NanoDrop 1000. 100 ng total RNA was dephosphorylated with calf intestinal phosphatase and then end-labeled with Cyanine-3-pCp using T4 RNA ligase. Labeled RNA was hybridized to the Agilent Technologies mouse miRNA microarray at 55 °C for 20 h. Slides were washed according to the Agilent Technologies miRNA protocol and scanned. Signals were extracted as described above.

### Flow cytometry analysis

Flow cytometry analysis, sorting and monoclonal antibody reagents, including CD11b (M1/70), CD40 (1C10), CD80 (16-10A1), CD86 (GL-1), CD38 [90], CD43 (S7), CD44 (IM7), CD21 (7G6), CD23 (B3B4), CD24 (30F1), CD27 (LG.3A10), CD49d (R1-2), Gr-1(RB6-8C5). Nod1^–/–^ mice were originally made [68], and generated in 4 mo ATAμκTg Nod1^+/–^ and Nod^–/–^ under C.B17 mice. Animal experiments were conducted under a protocol approved by the FCCC Institutional Animal Care and Use Committee (IACUC).

### Quantitative RT-PCR assay

Gene expression was quantitated by real-time PCR, using TaqMan assays from Applied Biosystems, an ABI 7500 real-time thermal cycler, and ABI software (Life Technologies). Relative gene expression levels were normalized using β-actin values for mRNA as a standard.

For spleen FO B versus pB1a in 2 mo, check of increased ZAP70 by anti-IgM, TNFa (aa80-235), DAP (C12-iE-DAP) (from InvivoGen), MDP (from InvivoGene). FO B (1 × 10^5^, 25λ) and pB1a cells (1 × 10^5^, 25λ) and together with anti-mouse IgM (15λ), DAP(15λ), MDP(15λ), TNFa (100 ng/ml, 15λ), then 20 h later, RT-PCR analysis with ZAP70.

### Intestinal microbiota

This intestinal microbiota positive analysis was published [25]. Crossreactivity of ATA IgM with intestinal microbiota as small intestine and large intestine as colon. For microbiota staining, fecal microbiota were prepared from C.B17.*scid* mouse intestine and incubated with 2 ug/ml IgM at room temperature for 60 min, followed by FL-anti IgM at 4 °C for 20 min, and then analyzed by flow cytometry. Monoclonal IgM (anti-KLH MM-30, BioLegend) was also used as a control.

### Heavy chain with surrogate light chain (SLC) association analysis

This was published for aMyIIA as negative and control IgH (SP6, V627) [25]. *IgH retrovirus production*: Selected IgH VDJ segments were amplified by PCR, cloned into an IgH-μ construct, and inserted into the pMIG retroviral vector (MSCV-IRES-GFP). Each IgH-pMIG plasmid and the pCL-Eco retroviral packaging vector were co-transfected into the Phoenix packing line and 24 h supernatant was collected, filtered, and stored at -80 °C until use. To analyze *Ig heavy chain associated with SLC (examine surrogate light-chain)*): Pro-B Abelson line N38 was transduced with IgH-pMIG retroviral supernatant. In GFP^+^ (green fluorescent protein) cells, IgH-μ transduction was examined by intracellular IgM staining, and extent of SLC assembly was examined by surface SLC staining, using the conformation-dependent anti-surrogate light-chain antibody SL156. To test the capacity for pre-BCR-mediated proliferation, IgH-pMIG retroviral supernatant was added to pro-B cell sultures of Rag1^–/–^ BALB/c BM on OP9 stromal cells together with IL-7. IgH (SP6, V627) as a positive control was set to 10.

### Supplementary Information

**Additional file 1: Figure S1.** Summary. In age, ATA B increased in PBL and CD5 can decrease, and increase of CD11b in ATA B, aMyIIA, and aPtC. V8-12 generation in MZ B cell.

**Additional file 2: Figure S2.** 2 mo wild time (WT) B1a cells versus ATAµκ Tg ATA B tumor.

**Additional file 3: Figure S3.** AGcA generation of NZ B and old aged generation of macrophages++ and intestinal tumor.

**Additional file 4. **

**Additional file 5. **

## Data Availability

No datasets were generated or analysed during the current study.
