# Supplementary material for: B-1 derived anti-Thy-1 B cells in old aged mice develop lymphoma/leukemia with high expression of CD11b and Hamp2 that different from TCL1 transgenic mice
Source: Immun Ageing. 2024 Apr 3;21:22. doi: 10.1186/s12979-024-00415-6 (PMC10988983; doi:10.1186/s12979-024-00415-6)
Supplement: Supplementary file 1 — Additional file 1: Figure S1. Summary. In age, ATA B increased in PBL and CD5 can decrease, and increase of CD11b in ATA B, aMyIIA, and aPtC. V8-12 generation in MZ B cell. [file 12979_2024_415_MOESM1_ESM.pdf]

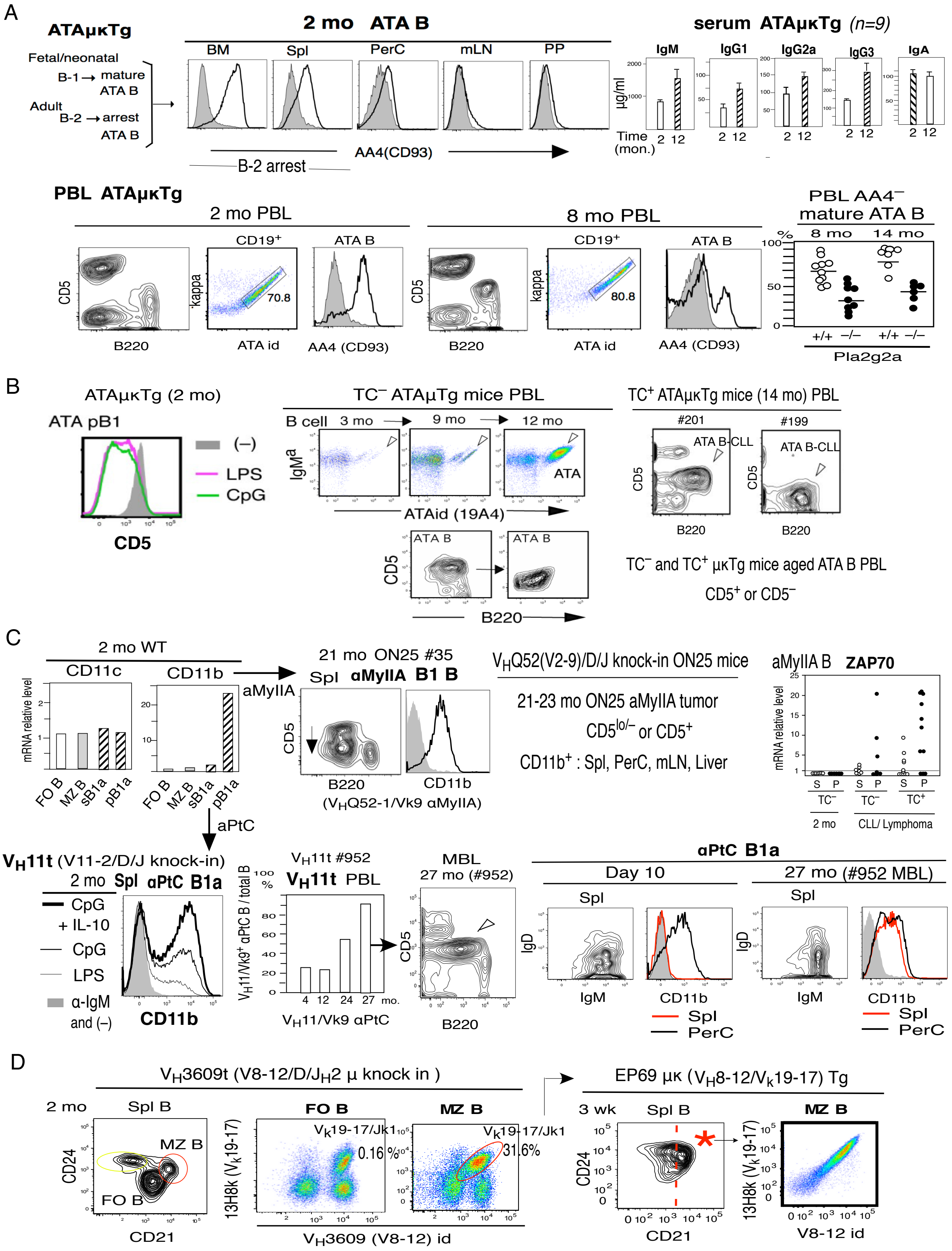

A
ATAμκTg
Fetal/neonatal
B-1 → mature ATA B
Adult
B-2 → arrest ATA B
2 mo ATA B
BM
Spl
PerC
mLN
PP
B-2 arrest
AA4(CD93)
serum ATAμκTg (n=9)
IgM
IgG1
IgG2a
IgG3
IgA
μg/ml
Time (mon.)
PBL ATAμκTg
2 mo PBL
8 mo PBL
CD19+
ATA B
CD5
B220
kappa
ATA id
70.8
80.8
AA4 (CD93)
PBL AA4− mature ATA B
8 mo
14 mo
%
Pla2g2a
B
ATAμκTg (2 mo)
ATA pB1
(−)
LPS
CpG
CD5
TC− ATAμTg mice PBL
B cell 3 mo
9 mo
12 mo
IgMa
ATA
ATAid (19A4)
ATA B
B220
TC+ ATAμκTg mice (14 mo) PBL
#201
#199
ATA B-CLL
TC− and TC+ μκTg mice aged ATA B PBL
CD5+ or CD5−
C
2 mo WT
CD11c
CD11b
mRNA relative level
FO B
MZ B
sB1a
pB1a
aMyIIA
21 mo ON25 #35
Spl αMyIIA B1 B
CD11b
(VHQ52-1/Vk9 αMyIIA)
VHQ52(V2-9)/D/J knock-in ON25 mice
21-23 mo ON25 aMyIIA tumor
CD5lo/− or CD5+
CD11b+ : Spl, PerC, mLN, Liver
aMyIIA B ZAP70
S P
TC−
TC+
2 mo
CLL/ Lymphoma
aPtC
VH11t (V11-2/D/J knock-in)
2 mo Spl αPtC B1a
CpG + IL-10
CpG
LPS
α-IgM and (−)
CD11b
VH11t #952
VH11t PBL
VH11/Vk9+ αPtC B / total B
VH11/Vk9 αPtC
mo.
MBL 27 mo (#952)
αPtC B1a
Day 10
27 mo (#952 MBL)
Spl
IgD
IgM
PerC
D
VH3609t (V8-12/D/JH2 μ knock in)
2 mo
Spl B
MZ B
FO B
CD24
CD21
FO B
MZ B
13H8k (Vk19-17)
Vk19-17/Jk1
0.16 %
31.6%
VH3609 (V8-12) id
EP69 μκ (VH8-12/Vk19-17) Tg
3 wk
V8-12 id

Figure S1

Figure S1. Summary. In age, ATA B increased in PBL and CD5 can decrease, and increase of CD11b in ATA B, aMyIIA, and aPtC. V8-12 generation in MZ B cell.
(A) 2 mo ATAμκTg mice with ATA B (V_H8-12/V_k21-5) show BM and most Spl with immature AA4$^+$, different most mature in PerC, mLN, and intestinal PP. In PBL, 2 mo ATA B cells are immature, then, 8 mo B1a cells increase with higher mature (AA4$^-$) ATA B cells (1), and further increase mature ATA B in 14 mo, contrust low ATA B cells in originally Pla2g2a negative ATAμκTg mice (1). Serum IgM, IgG1, IgG2, IgG3 also increase from 2 mo to 12 mo, and IgA originally 2 mo high. **(B)** Summary (2). Left: CD5 down-regulation after stimulation of ATA B pB1 cells in ATAμkTg mice by cell proliferation by LPS or CpG in 3 days. Middle: Increased ATA B cell frequency in PBL B cells in ATAμTg mouse at 12 mo, with further B220 down and CD5 reduction. Right: CD5 down-regulation occurrance at CLL stage also in TC$^+$ATAμκTg mice and CD5$^+$ and CD5$^-$ CLL in PBL found in two littermates at 14 mo. **(C)** Middle: aMyIIA B1 B cells showed CD5 down and CD11b$^+$ in old aged, summary of aMyIIA B (3). Right: ZAP70 expression determined by quantitative PCR in aMyIIA B cells from the spleen (s) and peritoneal cavity (p), after the tumor stage. Relative messenger RNA transcript levels with FO B was set to 1.0. Down: Summary of V_H11t (4). Left: Three days after indicated CD11b stimulation, LPS (low), CpG, CpG+IL-10 (high), of spleen B1a cells from an adult 2 mo V_H11t mice. Middle: In PBL, 12 mo aPtC B cells are 22 % and increased 27 mo 86.7% (#952) in aPtC (V_H11/V_k9) in CD19$^+$B cells as high MBL. Right: Comparison of CD11b expression levels by aPtC B1a cells in Spl between day 10 neonate as CD11b$^-$, and 27 mo as CD11b$^+$. **(D)** Summary about AGcA μTg and AGcA μκTg mice with MZ B (5). Left: Analysis of B cell subsets in the spleen. In FO B and MZ B cells, V_H3609 (V8-12) id$^+$ cells marked by a vertical line. The majority of 13H8K (V_k19-17/Jk1)$^{hi/med}$ Igk B cells are present in low FOB, and MZ B cells are higher. Right: AGcA μκTg mice, as EP69. 3 wk of EP69 μκTg mice showed dominantly in MZ B cells with the V_H8-12/V_k19-17.

1. Susan A.Shinton, Joni Brill-Dashoff, Kyoko Hayakawa. (2022). Pla2g2a promotes innate Th2-type immunity lymphocytes to increase B1a cells. Scientific Reports 12:14899

2. Hayakawa K, Formica AM, Brill-Dashoff J, Shinton SA, Ichikawa D, Zhou Y, Morse III HC, Hardy RR. (2016). Early generated B1 B cells with restricted BCRs become chronic lymphocytic leukemia with continued c-Myc and low Bmf expression. J. Exp. Med. 213:3007.

3. Hayakawa, K., Formica, A.M., Colombo, M.J., Shinton, S.A., Brill-Dashoff, J., Morse III, H.C., Li, Y.S., Hardy, R.R. (2016). Loss of a chromosomal region with syntheny to human 13q14 occurs in mouse chronic lymphocytic leukemia that originates from early-generated B-1 B cells. Leukemia 30:1510.

4. Hayakawa K, Formica A.M, Nakao Y, Ichikawa D, Shinton, S.A, Brill-Dashoff J, Smith, M.R, Morse III, H.C, Hardy, R.R. (2018). Early generated B-1 derived B cells have the capacity to progress to become mantle cell lymphoma-like neoplasia in aged mice. J. Immunol. 201: 804.

5. Ichikawa, D., Asano, A., Shinton, S.A., Brill-Dashoff, J., Formica, A.M., Velcich, A., Hardy, R.R., and Hayakawa K. (2015). Natural anti-intestinal goblet cell autoantibody production from Marginal zone B cells. J. Immunol. 194:606.
