# Supplementary material for: B-1 derived anti-Thy-1 B cells in old aged mice develop lymphoma/leukemia with high expression of CD11b and Hamp2 that different from TCL1 transgenic mice
Source: Immun Ageing. 2024 Apr 3;21:22. doi: 10.1186/s12979-024-00415-6 (PMC10988983; doi:10.1186/s12979-024-00415-6)
Supplement: Supplementary file 2 — Additional file 2: Figure S2. 2 mo wild time (WT) B1a cells versus ATAµκ Tg ATA B tumor. [file 12979_2024_415_MOESM2_ESM.pdf]

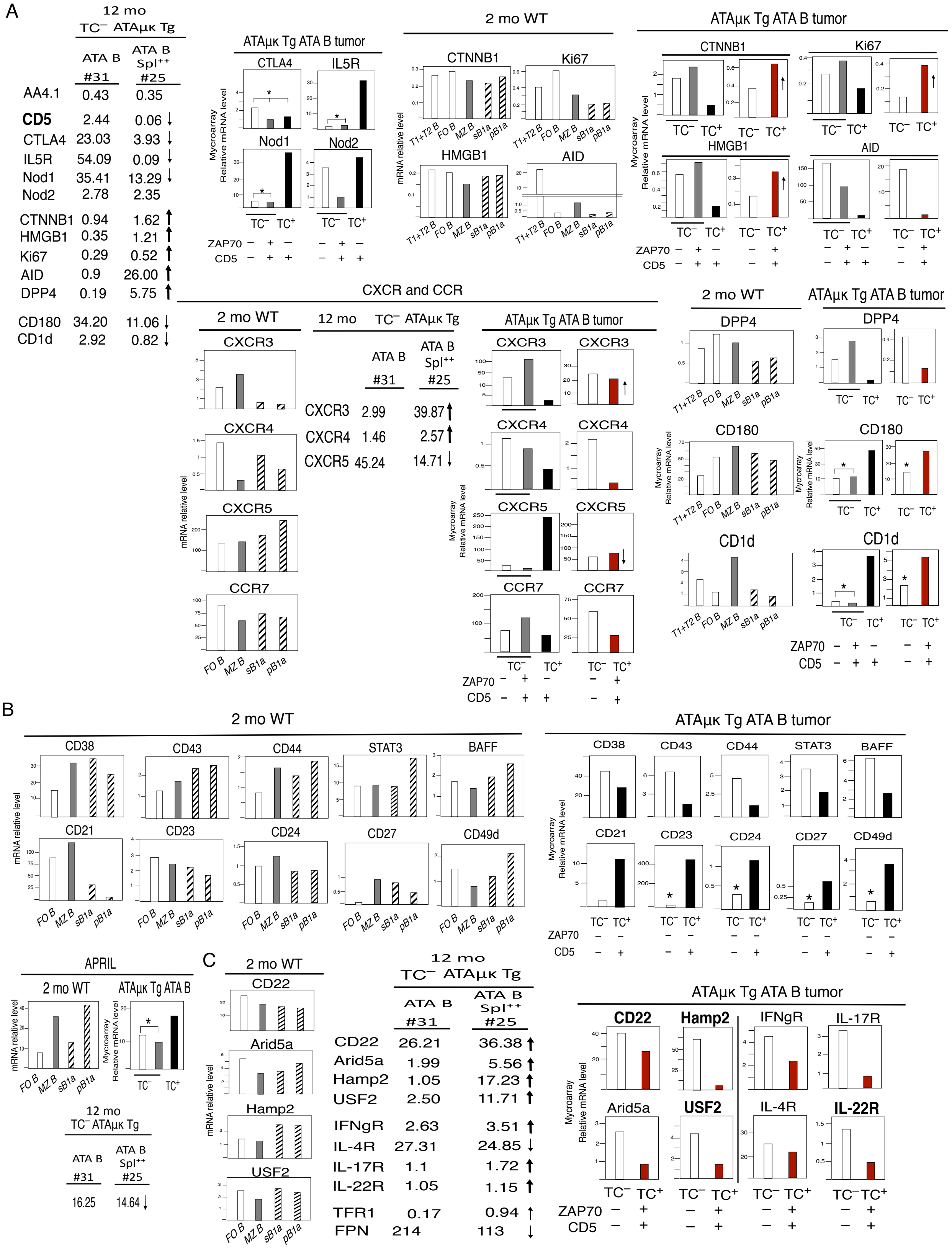
A
12 mo TC⁻ ATAμκ Tg
ATA B #31
ATA B Spl⁺⁺ #25
AA4.1 0.43 0.35
CD5 2.44 0.06 ↓
CTLA4 23.03 3.93 ↓
IL5R 54.09 0.09 ↓
Nod1 35.41 13.29 ↓
Nod2 2.78 2.35
CTNNB1 0.94 1.62 ↑
HMGB1 0.35 1.21 ↑
Ki67 0.29 0.52 ↑
AID 0.9 26.00 ↑
DPP4 0.19 5.75 ↑
CD180 34.20 11.06 ↓
CD1d 2.92 0.82 ↓
ATAμκ Tg ATA B tumor
CTLA4
IL5R
Nod1
Nod2
Mycoarray Relative mRNA level
TC⁻ TC⁺
ZAP70
CD5
2 mo WT
CTNNB1
Ki67
HMGB1
AID
mRNA relative level
T1+T2 B
FO B
MZ B
sB1a
pB1a
CXCR and CCR
CXCR3
CXCR4
CXCR5
CCR7
CXCR3 2.99 39.87 ↑
CXCR4 1.46 2.57 ↑
CXCR5 45.24 14.71 ↓
DPP4
CD180
CD1d
B
CD38
CD43
CD44
STAT3
BAFF
CD21
CD23
CD24
CD27
CD49d
APRIL
ATAμκ Tg ATA B
16.25 14.64 ↓
C
CD22
Arid5a
Hamp2
USF2
CD22 26.21 36.38 ↑
Arid5a 1.99 5.56 ↑
Hamp2 1.05 17.23 ↑
USF2 2.50 11.71 ↑
IFNgR 2.63 3.51 ↑
IL-4R 27.31 24.85 ↓
IL-17R 1.1 1.72 ↑
IL-22R 1.05 1.15 ↑
TFR1 0.17 0.94 ↑
FPN 214 113 ↓
IFNgR
IL-17R
IL-4R
IL-22R

Figure S2

Figure S2. 2 mo wild time (WT) B1a cells versus ATAμκ Tg ATA B tumor.

2 mo WT B cells: spleen-immature B (T1+T2 B), FO B, MZ B, and spleen B1a (sB1a,) and PerC B1a (pB1a) in mRNA. ATA B tumor for mycloarray analysis mRNA: as listed Figure 4A, TC^{-}ZAP70^{-}CD5^{-} (16 mo) and TC^{-}ZAP70^{+}CD5^{+} (22 mo), versis middle aged ATA B tumor TC^{+}ZAP70^{-}CD5^{+} (10 mo). Figure 4B, TC^{-}ZAP70^{-}CD5^{-} (17 mo) versus TC^{+}ZAP70^{+}CD5^{+} (15 mo). As in Figure 5A, 12 mo TC^{-}ATAμκTg mice ATA B with normal CD5^{+}B #31, and CD5^{-}B with spleen increased #25. **(A)** 12 mo ATA B for TC^{-} cells, CD5^{+} #31 showed slightly AA4.1 (immature) present, however most mature B1a, and #25 is the mature CD5^{-} B cell with Spl^{++}. This 12 mo CD5^{-} ATA B cell is similar to old aged TC^{-}ATA B tumors, down IL5R and Nod1, increase CTNNB1, HMGB1, Ki67, AID and DPP4, down CD180 and CD1d, as TC^{-} compared to middle aged TC^{+}ZAP70^{-}CD5^{+}. However, both TC^{-} and TC^{+}ZAP70^{-} cells are CTLA4 down. Nod2 is TC^{-}ZAP70^{+}CD5^{+} cells are down. CTNNB1, HMGB1, Ki67 are higher in TC^{-} ZAP70^{+}CD5^{+} than TC^{-}ZAP70^{-}CD5^{-} and similar to high TC^{+}ZAP^{+}CD5^{+}. AID was originally (in 2 mo) all mature B cells were low, then, old aged TC^{-}ZAP^{-}CD5^{-}ATA B tumor more increased than TC^{-}ZAP70^{+}CD5^{+} cells and TC^{+} cells are negative. Down: **For CXCR**. CXCR5 was originally high in B1a cells, then, 12 mo CD5^{-} spl^{++} cell showed down CXCR5, then CXCR4 and CXCR3 increased as old aged TC^{-}ATA B tumor. TC^{+}ZAP70^{-}CD5^{+} cells are continuously CXCR5^{+} and low CXCR4 and CXCR3. TC^{+}ZAP70^{+}CD5^{+} cells are lower CXCR5 but low CXCR4 and slightly increased CXCR3. CCR7 is slightly higher by old aged TC^{-} cells than TC^{+}. **(B)** 2 mo WT B cells compared with TC^{-}ZAP70^{-}CD5^{-} versus TC^{+}ZAP70^{-}CD5^{+} in listed Figure 4A1,3. 2 mo WT CD38, CD43, CD44, STAT3, BAFF are higher in pB1a, and old age TC^{-} ATA B are more increased than middle aged TC^{+}, but TC^{+}ZAP70^{+}CD5^{+} increased for CD44 and STAT3 (Figure 4B2). TC^{+} in CD21, CD23. CD24, CD27, CD49d are higher than TC^{-}. In 2 mo WT B1a cells, CD21 negative and CD23 low originally, and low in TC^{-} and TC^{+}ZAP70^{+}CD5^{+} also low. CD49d showed higher in original pB1a, then all CD24, CD27, CD49d were low in old aged TC^{-}ATA B tumor (both ZAP70^{-} and ZAP70^{+}) than TC^{+}. Since CD27 is generally lower in all B cells in mice (not human) TC^{+}Tg slightly increased. CD49d and CD24 down are old aged TC^{-}ATA B tumor. In APRIL, 2 mo WT showed higher in pB1a and TC^{-}CD5^{-}showed decreased at 12 mo and also low in old aged TC^{-}ATA B tumor, compared to increased BAFF. **(C)** 2 mo WT, CD22 and Arid5a are FOB > B1a, and Hamp2 are FOB < B1a. Then, 12 mo TC^{-}CD5^{-} cells showed similar to old aged TC^{-} ATA B tumor than middle aged TC^{+}ZAP70^{-}CD5^{+} cells (Figure 5C) and also TC^{+}ZAP70^{+}CD5^{+} cells. Clearly, high CD22R and Arid5a, and higher Hamp2, USF2^{+}, and IL-22R^{+} in TC^{-} than TC^{+}(ZAP70^{-} and ZAP70^{+}). Thus, not Hamp2 increased in TC^{+}Tg mice.
