# Supplementary material for: B-1 derived anti-Thy-1 B cells in old aged mice develop lymphoma/leukemia with high expression of CD11b and Hamp2 that different from TCL1 transgenic mice
Source: Immun Ageing. 2024 Apr 3;21:22. doi: 10.1186/s12979-024-00415-6 (PMC10988983; doi:10.1186/s12979-024-00415-6)
Supplement: Supplementary file 3 — Additional file 3: Figure S3. AGcA generation of NZ B and old aged generation of macrophages++ and intestinal tumor. [file 12979_2024_415_MOESM3_ESM.pdf]

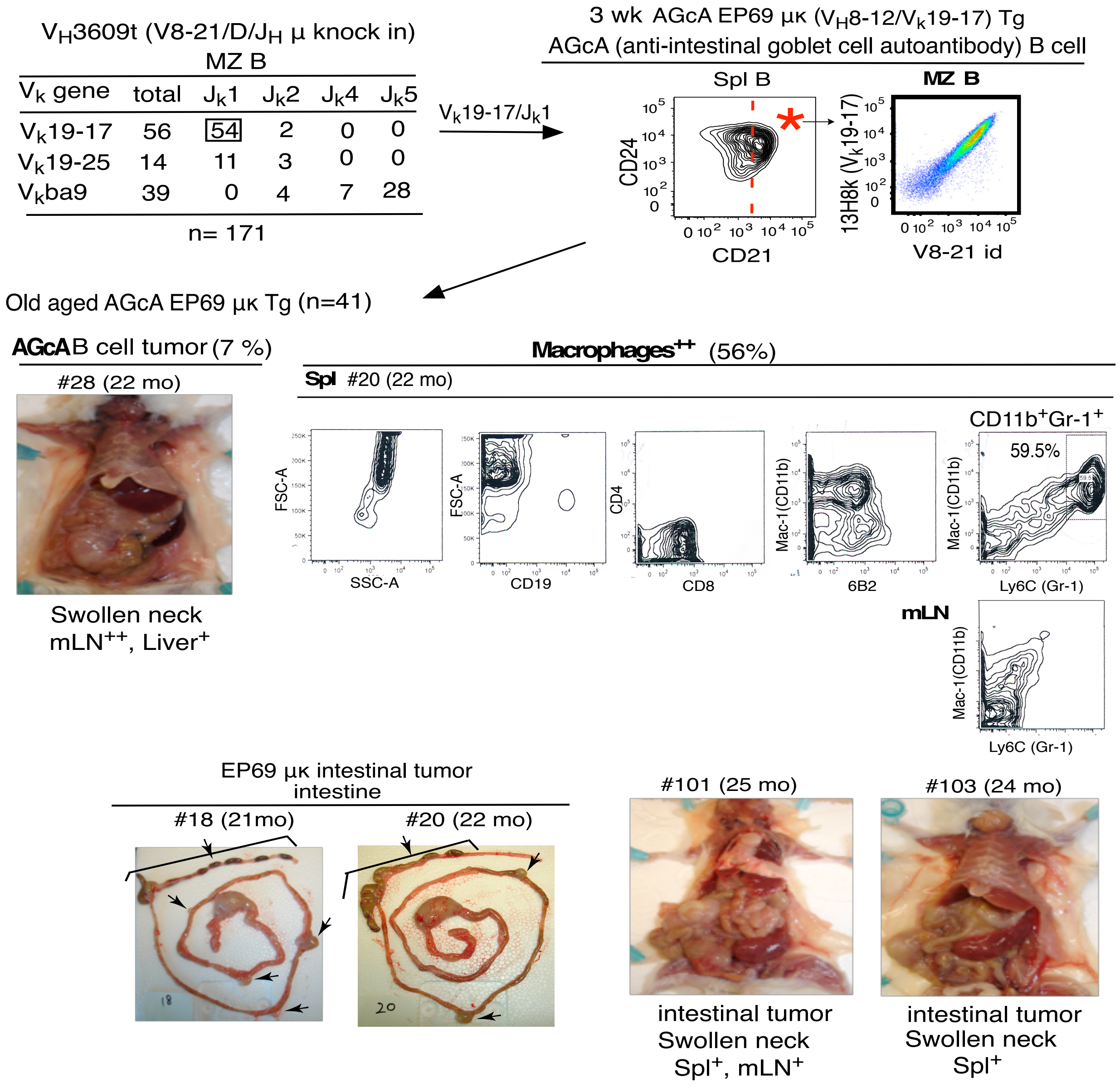

Figure S3. AGcA generation of NZ B and old aged generation of macrophages++ and intestinal tumor. Summary of V_H3609t (V8-12/D/JH knock in) in MZ B cells with higher V_k19-17/J_k1. V8-12/V_k19-17 B cell is the AGcA (a natural anti-intestinal goblet cell autoantibodiy), and AGcA μκTg (EP69 μκTg) generate higher MZ B cells with V8-12/V_k19-17 (1). In this EP69 μκTg mice, old aged 7% generated AGcA B cell tumor (mLN^+, $Liver^+$), and 56% by $Macrophage^{++}$ ($CD11b^+Gr\text{-}1^+$) higher in spleen with probably increased iron, with intestinal tumor generation as seen in small intestine (not high macrophage) and more in large intestine (colon). Also, a swollen lymph node increase with in neck.

1. Ichikawa, D., Asano, A., Shinton, S.A., Brill-Dashoff, J., Formica, A.M., Velcich, A., Hardy, R.R., and Hayakawa K. (2015). Natural anti-intestinal goblet cell autoantibody production from Marginal zone B cells. J. Immunol. 194:606.
