# Supplementary material for: B-1 derived anti-Thy-1 B cells in old aged mice develop lymphoma/leukemia with high expression of CD11b and Hamp2 that different from TCL1 transgenic mice
Source: Immun Ageing. 2024 Apr 3;21:22. doi: 10.1186/s12979-024-00415-6 (PMC10988983; doi:10.1186/s12979-024-00415-6)
Supplement: Supplementary file 4 — Additional file 4. [file 12979_2024_415_MOESM4_ESM.pdf]

Figure S4-1 ($TC^{-}ZAP70^{+}CD5^{+}$ and $TC^{+}ZAP70^{+}CD5^{+}$ high)

TNFa in CLL: Foa R., Massaia, M., *et al* (1990). Production of tumor necrosis factor-alpha by B-cell chronic lymphocytic leukemia cells: A possible regulatory role of TNF in the progression of the disease. *Blood* 76:393

CD40 higher in U-CLL: Haselager, M.V., Kater, A.P., Eldering, E. (2020). Proliferative signals in chronic lymphocytic leukemia; What are we missing ?. *Fronters in Oncology*10:592205

IL-4 in U-CLL than M-CLL: Guo, B., Zhang,L., *et al* (2016). IL-4 rescues surface IgM expression in chronic lymphocytic leukemia. *Blood* 128:553

Cyclin D1 a nall subset of CLL: Abboudi, Z., Patel, K., Naresh, K.N. (2009). Cyclin D1 expression in typical chronic lymphocytic leukaemia. *Eur J Haematol* 83:203

Cyclin E in CLL: Decker, T., Hipp, S., *et al* (2004). Expression of cyclin E in resting and activated B-chronic lymphocytic leukaemia cells: cyclin E/cdk2 as a potential therapeutic target. *Br J Haematol* 125:141

Tubb in CLL: Fressato, F., Trimarco, V., *et al* (2014). Leukaemic cells from chronic lymphocytic leukaemia patients undergo apoptosis following microtubule depolymerization and Lyn inhibition by nocodazole. *Br J Haematol* 165:659

cMyc, higher in U-CLL than M-CLL. Yeomans, A., Thirdborough, S.M., *et al* (2016) Engagement of the B-cell receptor of chronic lymphocytic leukemia cells drives global and MYC-specific mRNA transiation. *Blood* 127:449

Fzd2 in $ZAP70^{+}$CLL: Griffen, T.L., Dammer, E.B., *et al* (2021). Multivariate transcriptome analysis identifies networks and key drivers of chronic lymphocytic leukemia relapse risk and patient survival. *BMC Med Genomics* 14:171

Ki67 high in $ZAP70^{+}$ U-CLL: Mittal, A.K., Chaturvedi, N.K., *et al* (2013). Role of CTLA4 in the proliferation and survival of chronic lymphocytic leukemia. *Plos one* 8:e70352.

CD86: only small portion of the CLL cells: Mallm, J-P, Iskar, M., *et al* (2019). Linking aberrant chromatin features in chronic lymphocytic leukemia to transcription factor networks. *Molecular Systems Biology* 15:e8339

Figure S4-2 (TC^{-} and $TC^{+}ZAP70^{+}$ high, and $TC^{-}>TC^{+}$)

Cyclin D2 in CLL: Decker,T., Schneller, F., *et al* (2002) Cell cycle progression of chronic lymphocytic leukemia cells is controlled by cyclin D2, cyclin D3, cyclin-dependent kinase(cdk) 4 and the cdk inhibitor p27. *Leukemia* 16:327

TLR7 in CLL, and U-CLL high versus M-CLL: Spaner, D.E., Shi, Y., *et al* (2006). Immunomudulatory effects of Toll-like receptor-7 activation on chronic lymphocytic leukemia cells. *Leukemia* 20:286
Ntoufa, S., Varadi, A., *et al* (2012), Distinct innate immunity pathways to activation and tolerance in subgroups of chronic lymphocytic leukemia with distinct immunoglobulin receptors. *Mol Med* 18:1281

Wnt10a,b in CLL: Janovska, P., Bryla, V. (2017). Wnt signaling pathways in chronic lymphocytic leukaemia and B-cell lymphomas. *British Journal of Pharmacology.* 174:4701
Lu, D., Zhao, Y., *et al* (2004). Activation of the Wnt signaling pathway in chroni lymphocytic leukemia. *PNAS* 101:3118
Wu, Q., Claudia, Z. Ranheim, E.A. (2007). Dysregulation of frizzled 6 is a critical componen of B cell leukemogenesis in a mouse model of chronic lymphocytic leukemia. *Blood* 110:347

ERK2 in CLL: Burger, J.A., Tsukada, N., *et al* (2000). Blood-derived nurse-like cells protect chronic lymphocytic leukemia B cells from spontaneous apoptosis through stromal cell-derived factor-1. *Blood* 96:2655

Myd88 in CLL: Martinez-Trillos, A., Pinyol, M. *et al* (2014) Mutations in TLR/MYD88 pathway identify a subset of young chronic lymphocytic leukemia patients with favorable outome. *Blood* 123:3790
Skorka, K., Wlasiuk, P., *et al* (2021) Aberrant expression of TLR2, YLR7, TLR9, splicin variants of TLR4 and MY88 in chronic lymphocytic leukemia patients. *J Clin Med* 10:867

STAT3 in CLL: Ma, W., Zhang, Y., *et al* (2019). STAT3 promotes chronic lymphocytic leukemia progression through upregulating SMYD3 expression. *Arch Med Sci* 15:1163

IL-10 in CLL: Drennan, S., D'Avola, A., *et al* (2017). IL-10 production by CLL cells is enhanced in the anergic IGHV mutated subset and associates with reduced DNA methylation of the IL10 locus. *Leukemia* 31:1686

IL-6 in CLL: Fayad, L., Keating, M.J, *et al* (2001). Interleukin-6 and interleukin-10 levels in chronic lymhocytic leukemia: correlation with phenotypic characteristics and outcome. *Blood* 97:256
Buggins, AGS, Pattern, PEM, *et al* (2008). Tumor-derived IL-6 may contribute to the immunological defect in CLL. *Leukemia* 22:1084

CD44 high in U-CLL: Zhang, S., Wu, C.C.N, *et al* (2013) Targeting chronic lymphocytic leukemia cells with a humanized monoclonal antibody specific for CD44. *PNAS* 110:6127

Ki67 high in ZAP70+ U-CLL: Mittal, A.K., Chaturvedi, N.K., *et al* (2013). Role of CTLA4 in the proliferation and survival of chronic lymphocytic leukemia. *Plos one* 8:e70352.

CTNNB1 in CLL: Sinha, S., Secreto, C.R., *et al* (2021). Upregulation of AXL and β-catenin in chronic lymphocytic leukemia cells cultured with bone marrow stroma cells is associated with enhanced drug resistance. *Blood Cancer Journal* 11:37

HMGB1 in CLL plasma high: Jia, L., Clear, A., *et al* (2014) Extracellular HMGB1 promotes differentiation of nurse-like cells in chronic lymphocytic leukemia. *Blood* 123:1709

TLR9 in CLL: Spaner, DE, Masellis A. (2007). Toll-like receptor agonists in the treatment of chronic lymphocytic leukemia. *Leukemia* 21:53

Wnt1 mutation found: Janovska',P, Bryja,V. (2017). Wnt signaling pathways in chronic lymphocytic leukaemia and B-cell lymphoma. *British Journal of Phamacology* 174:4701

Fzd1 and Fzd5: Wnt10a-Fzd1/Fzd10, Wnt10b-Fzd5: Chen,H, Wang, Y, Xue, F. (2013). Expression and the clinical significance of Wnt10a and Wnt10b in endometrial cancer are associated with the Wnt/b-catenin pathway. *Oncology reports* 29:507

LRP6: Wnt10b-LRP6: Karczewska-Kupczewska, M., Stefanowiz, M, *et al* (2016). Wnt signaling genes in adipose tissue and skeletal muscle of humans with different degrees of insulin sensitivity. *J Clin Endocrinal Metab* 101:3079

Dock2, Rac1/2 in CLL: Hasan, MK, Yu, J., *et al* (2018). Wnt5a induces ROR1 to recruit DOCK2 to activate Rac1/2 in chronic lymphocytic leukemia. *Blood* 132:170

Bcl2 high in CLL: Majid, A., Tsoulakis, O., *et al* (2008). Bcl2 expression in chronic lymphocytic leukemia: lack of association with the Bcl2-938A>C promoter single nucleotide polymorphism. *Blood* 111:874

BAFF in CLL: Endo, T., Nishio, M. *et al* (2007). BAFF and APRIL support chronic lymphocytic leukemia B-cell survival through activation of the canonical NF-kB pathway. *Blood* 109:703

CD38 high in U-CLL: Damie, R.N., Wasil, T., *et al* (1999). Ig V gene mutation status and CD38 expression as novel prognostic indicators in chronic lymphocytic leukemia. *Blood* 94:1840

CD43 in CLL: Sorigue, M., Junca, J., *et al* (2018). Expression of CD43 in chronic lymphoproliferative leukemias. *Cytometry B Clin Cytom* 948:136

CD80 in CLL: Do, P., Beckwith, K.A., *et al* (2019). Leukemic B cell CTLA-4 suppresses costimulation of T cells. *J Immunol* 202:2806

Tubb in CLL: Reichle A., Diddens, H., *et al* (1995). Beta-Tubulin and P-glycoprotein: Major determinants of vincristine accumulation in B-CLL cells. *Leukemia Research* 19:823.
Fressato, F., Trimarco, V., *et al* (2014). Leukaemic cells from chronic lymphocytic leukaemia patients undergo apoptosis following microtubule depolymerization and Lyn inhibition by nocodazole. *British Journal of Haematology* 165:659

T-bet in CLL: Dorfman, DM, Hwang, ES., *et al* (2004). T-bet, a T-cell-associated transcription factor, is expressed in a subset of B-cell lymphoproliferative disorders. *Am J Clin Pathol* 122:292

CD11c in CLL: Umit, E.G., Baysal, M., *et al* (2017). CD11c expression in chronic lymphocytic leukemia revisited, related with complications and survival. *Int J Lab Hematol* 39:552
Newman, R.A, Peterson, B., *et al* (1993). Phenotypic markers and Bcl-1 gene rearrangement in B-cell chronic lymphocytic leukemia: a cancer and leukemia group B study. *Blood* 82:1239

CD11b low in CLL: Newman, R.A., Peterson, B., *et al* (1993). Phenotypic markers and Bcl-1 gene rearrangements in B-cell chronic lymphocytic leukemia: A cancer and leukemia group B study. *Blood* 82:1239

CXCR4 in CLL/U-CLL: Ponader, S., Chen, S-S, *et al* (2012). The bruton tyrosine kinase inhibitor PCI-32765 thwarts chronic lymphocytic leukemia cell survival and tissue homing in vitro and in vivo. *Blood* 119:1182
Ganghammer, S., Gutjahr, J., *et al* (2016). Combined CXCR3/CXCR4 measurements are of high prognostic value in chronic lymphocytic leukemia due to negative co-operativity of the ereceptors. *Haematologica* 101:e99
McCaig, A.M., Cosimo, E., *et al* (2012). Dasatinib inhibits CXCR4 signaling in chronic lymphocytic leukaemia cells and impaires migration towards CXCL12. *Plos one* 7:e48929.

DPP4 (CD26) in CLL and serum high in U-CLL: Bauvois, B., Meester, I.D, *et al* (1999). Constitutive expression of CD26/dipeptidylpeptidase IV on peripheral blood B lymphocytes of patients with B chronic lymphocytic leukaemia. *British Journal of Cancer* 79:1042
Molica, S., Digiesi, G., *et al* (2009). Serum levels of CD26 predicts time to first treatment in early B-chronic lymphocytic leukemia. *Eur J Haematol* 83:208

AID high in U-CLL than M-CLL: Oppezzo, P., Navarrete, M., Chiorazzi, N. (2021). AID in chronic lymphocytic leukemia: induction and action during disease progression. *Frontiers in Oncology* 11:634383

Palacios, F., Moreno, P, *et al* (2010). High expression of AID and active class switch recombination might account for a more aggressive disease in unmutated CLL patients: link with an activated microenvironment in CLL disease. *Blood* 115:4488

Figure S4-3 (TC$^-$ down, and TC$^+$ > TC$^-$)

CD5$^+$CLL, and low CD5$^-$CLL: Cartron, G., Linassier, C., *et al* (1998). CD5 negative B-cell chronic lymphocytic leukemia: clinical and biological features of 42 cases. *Leuk Lymphoma* 31:209

Huang, J.C., William G., *et al* (1999). CD5$^-$ small B-cell leukemias are rarely classifiable as chronic lymphocytic leukemia. *Am J Clin Paathol* 111:123

Nod1+ in CLL, can be down: Musio, M.,Scielzo, C., *et al* (2009). Expression and function of toll like receptors in chronic lymphocytic leukaemia cells. *Br J Haematol* 144:507

IL-5$^+$, lack of IL-5 can occur in CLL: Hayes, T.G., Tan, X.L., *et al* (1993). Abnormal response to IL-5 in B-cell chronic lymphocytic leukemia. *Leuk Res* 17:777

CTLA4$^+$ in CLL, and down in CLL: Mittal, A.K., Chaturvedi, N.K., *et al* (2013). Role of CTLA4 in the proliferation and survival of chronic lymphocytic leukemia. *Plos one* 8:e70352

TLR1 and TLR6 in CLL: Muzio, M., Scielzo, C., *et al* (2008). Expression and function of toll like receptors in chronic lymphocytic leukaemia cells. *British Journal of Haematology*144:507

TLR2 in CLL: Skorka, K., Wlasiuk, P., *et al* (2021). Aberrant expression of TLR2, TLR7, TLR9, splicing variants of TLR4 and Myd88 in chronic lymphocytic leukemia patients. *J Clin Med* 10:867

TLR4, low in CLL: Rybka, J., Butrym, A., *et al* (2016). The expression of Toll-like receptors in patients with B-cell chronic lymphocytic leukemia. *Arch Immunol Ther Exp* 64:S147

Wnt4, in CLL: Memarian, A, Hojjat-Farsangi, M,*et al* (2009). Variation in WNT genes expression in different subtypes of chronic lymphocytic leukemia. *Leuk Lymphoma* 50:2061

Wnt5b in U-CLL than M-CLL: Suthon, S., Perkins, R.S., *et al* (2021). Wnt5b in physiology and disease. *Frontiers in Cell and Developmental Biology* 9:667581

Wnt 6 in CLL: Lu D., Zhao,Y., *et al* (2004). Activation of the Wnt signaling pathway in chronic lymphocytic leukemia. *PNAS* 101:3118

Wnt11 positive, but not increast in CLL: Lu, D, Zhao, Y,*et al* (2004). Activation of the Wnt signaling pathway in chronic lymphocytic leukemia. *Proc Natl Acad Sci U S A* 101:3118

Fzd6 in U-CLL: Corda, G., Sala, A. (2017). Non-canonical WNT/PCP signalling in cancer: Fzd6 takes centre stage. *Oncogenesis* 6:e364.

Dahiya, S., Saini, V., *et al* (2019). Insights into molecular interactions of human Wnt5b and frizzled proteins for their role in teratogenicity. *Bioinformation* 15:246

ROR2 in U-CLL (also ROR1): Suthon, S., Perkins, R.S., *et al* (2021). Wnt5b in physiology and disease. *Frontiers in Cell and Developmental Biology* 9:667581

Rac2 in CLL: Hasan, M.K., Yu, J., *et al* (2018). Wnt5a induces ROR1 to recruit DOCK2 to activate Rac1/2 in chronic lymphocytic leukemia. *Blood* 132:170

ERK1 in CLL: Burger, J.A., Tsukada, N., *et al* (2000). Blood-derived nurse-like cells protect chronic lymphocytic leukemia B cells from spontaneous apoptosis through stromal cell-derived factor-1. *Blood* 96:2655.

Hasan, M.K., Yu, J., *et al* (2018). Wnt5a induces ROR1 to recruit DOCK2 to activate Rac1/2 in chronic lymphocytic leukemia. *Blood* 132:170

Runx2 from Wnt5, and LEF1 interacts with Runx2: Arnsdorf, E., Tummala, P., Jacobs, C.R.(2009). Non-canonical Wnt signaling and N-cadherin related β-catenin signaling play a role in mechanically induced osteogenic cell fate. *Plos one* 4:e5388

Komori, T., (2011). Signaling networks in Runx2-dependent bone development. *J Cell Biochem* 112:750

NFKB1 activated in CLL (unmulated CLL): Mansouri, L., Papakonstantinou, N., *et al* (2016). NF-kB activation in chronic lymphocytic leukemia: A point of convergence of external triggers and intrinsic lesions. *Seminars in Cancer Biology* 39:40

Gaudio, E., Spizzo, R., *et al* (2012). Tcl1 interacts with Atm and enhances NF-kB activation in hematologic malignancies. *Blood* 119:180

P300 in CLL: Rozovski, U., Harris, D.M., *et al* (2018). STAT3 is constitutively acetylated on lysine 685 residues in chronic lymphocytic leukemia cells. *Oncotarget* 9:33710

Bhatt, D., Ghosh, S. (2014). Requlation of the NF-kappaB-mediated transcription of inflammatory genes. *Front Immunol* 5:71

April in CLL: Endo, T., Nishio, M., *et al* (2007). BAFF and APRIL support chronic lymphocytic leukemia B-cell survival through activation of the canonical NF-kB pathway. *Blood* 109:703

LRP5/6 in CLL: Staal, F.J.T., Famili, F., *et al* (2016). Aberrant Wnt signaling in leukemia. *Cancers* 8:78

LEF1 in CLL: Erdfelder, F., Hertweck, *et al* (2010). High lymphoid enhancer-binding factor-1 expression is associated with disease progression and poor prognosis in chronic lymphocytic leukemia. *Hematology Reports* 2:e3

Wu,W., Zhu, H., *et al* (2016). High LEF1 expression predicts adverse prognosis in chronic lymphocytic leukemia and may be targeted by ethacynic acid. *Oncotarget* 7:21631

CD21 low can in U-CLL: Nichols, E-M., Jones, R., *et al* (2015). A CD21 low phenotype, with no evidence of autoantibodies to complement proteins, is consistent with a poor prognosis in CLL. *Oncotarget* 6:32669

CD23+ in CLL but CD23low occur: Barna, G., Reiniger, L., *et al* (2008). The cut-off levels of CD23 expression in the differential diagnosis of MCL and CLL. *Hematol Oncol* 26:167

CD24 in CLL: Christian, S.L. (2022). CD24 as a potential therapeutic target in patients with B- cell leukemia and lymphoma: current insights. *OncoTargets and Therapy* 15:1391

Darwiche, W., Bubler B., *et al* (2018). Chronic lymphocytic leukemia B-cell normal cellular counterpart: Clues from a functional perspective. *Frontiers in Immunology* 9:683

CD27 in CLL and $CD27^{-}$ in U-CLL: Tanheim, EA, Cantwell MJ, Kipps, TJ. (1995). Expression of CD27 and its ligand, CD70, on chronic lymphocytic leukemia B cells. *Blood* 85:3556

Seifert, M., Sellmann, L, *et al* (2012). Cellular origin and pathophysiology of chronic lymphocytic leukemia. *J Exp Med* 209:2183

CD49d half positive in CLL, and aged some CD49d reduced: Kunnumbrath, A., Singh, N., *et al* (2022). Flow cytometric expression of CD49d in newly diagnosed chronic lymphocytic leukemia and its correlation with established prognostic markers. *J Lab Physicians* 14:435.

CXCR5 in CLL, CXCR4 > CXCR5: Ponader, S., Chen, S-S, *et al* (2012). The bruton tyrosine kinase inhibitor PCI-32765 thwarts chronic lymphocytic leukemia cell survival and tissue homing in vitro and in vivo. *Blood* 119:1182

CD1d low in CLL: Zheng, Z., Venkatapathy, S., *et al* (2002). Expression profiling of B cell chronic lymphocytic leukemia suggests deficient CD1-mediated immunity, polarized cytokine response, altered adhesion and increased intracellular protein transport and processing of leukemic cells. *Leukemia* 16:2429

Kotsianidis, L., Nakou, E., *et al* (2011). The diagnostic value of CD1d expression in a large cohort of patients with B-cell chronic lymphoproliferative disorders. *Am J Clin Pathol* 136:400

$CD180^{-}$ in U-CLL than $CD180^{+}$ in M-CLL: Porakishvili, N., Kulikova, N., *et al* (2005). Differential expression of CD180 and IgM by B-cell chronic lymphocytic leukaemia cells using mutated and unmutated immunoglobulin VH genes. *British J Haematology* 131:313.
