# Supplementary material for: B-1 derived anti-Thy-1 B cells in old aged mice develop lymphoma/leukemia with high expression of CD11b and Hamp2 that different from TCL1 transgenic mice
Source: Immun Ageing. 2024 Apr 3;21:22. doi: 10.1186/s12979-024-00415-6 (PMC10988983; doi:10.1186/s12979-024-00415-6)
Supplement: Supplementary file 5 — Additional file 5. [file 12979_2024_415_MOESM5_ESM.pdf]

Figure S5

Breast Cancer

Thy-1 (ECs/MSC): McLean, K, Gong, Y, *et al* (2011). Human ovarian carcinoma-associated mesenchymal stem cells regulate cancer stem cells and tumorigenesis via altered BMP production. J Clin Invest 121:3206
Brenet, M, Martinez, S, *et al* (2020). Thy-1 (CD90)-Induced Metastatic Cancer Cell Migration and Invasion Are beta3 Integrin-Dependent and Involve a Ca(2+)/P2X7 Receptor Signaling Axis. Front Cell Dev Biol 8:592442

Hepcidin^{+} iron down: Schmidt, P. J. (2015). Regulation of Iron Metabolism by Hepcidin under Conditions of Inflammation. J Biol Chem 290:18975

EMT-CD44^{hi} CD24^{low}: Tsai, J. H, Yang, J. (2013). Epithelial-mesenchymal plasticity in carcinoma metastasis. Genes Dev 27:2192
Al-Hajj, M, Wicha, M. S, *et al* (2003). Prospective identification of tumorigenic breast cancer cells. Proc Natl Acad Sci U S A 100:3983

CTNNB1^{+}: Geyer, F. C, Lacroix-Triki, M, *et al* (2011). beta-Catenin pathway activation in breast cancer is associated with triple-negative phenotype but not with CTNNB1 mutation. Mod Pathol 24:209

HMGB1^{+}:Sun, S, Zhang, W, *et al* (2015). High mobility group box-1 and its clinical value in breast cancer. Onco Targets Ther 8:413

Ki67^{+}: Soliman, N. A, Yussif, S. M. (2016). Ki-67 as a prognostic marker according to breast cancer molecular subtype. Cancer Biol Med 13:496

CXCR4^{+}: Mukherjee, D, Zhao, J. (2013). The Role of chemokine receptor CXCR4 in breast cancer metastasis. Am J Cancer Res 3:46

DPP4 (CD26)$^{++}$ in serum: Eric-Nikolic, A, Matic, I. Z, *et al* (2011). Serum DPPIV activity and CD26 expression on lymphocytes in patients with benign or malignant breast tumors. Immunobiology 216:942

Pancreatic Cancer

Thy-1 (Fibroglasts, ECs (endo): Zhu, J, Thakolwiboon, S, *et al* (2014). Overexpression of CD90 (Thy-1) in pancreatic adenocarcinoma present in the tumor microenvironment.
PLoS One 9:e115507

Hepcidin ^{low} iron over: Brissot, P, Pietrangelo, A, *et al* (2018). Haemochromatosis. Nat Rev Dis Primers 4:18016

CD44^{+}CD24^{+}: Li, C, Heidt, D. G, *et al* (2007). Identification of pancreatic cancer stem cells. Cancer Res 67:1030

EBT-CD44^{+}CD24^{-}: Peng, L, Liu, Z, *et al* (2017). MicroRNA-148a suppresses epithelial-mesenchymal transition and invasion of pancreatic cancer cells by targeting Wnt10b and inhibiting the Wnt/beta-catenin signaling pathway. Oncol Rep 38:301

CTNNB1^{+} mutation: Saki, K, Mansouri, V, *et al* (2021). Common and differential features of liver and pancreatic cancers: molecular mechanism approach. Gastroenterol Hepatol Bed Bench 14:S87

HMGB1^{+} : Cebrian, M. J, Bauden, M, *et al* (2016). Paradoxical Role of HMGB1 in Pancreatic Cancer: Tumor Suppressor or Tumor Promoter?. Anticancer Res 36:4381

Ki67^{+}: Hamilton, N. A, Liu, T. C, *et al* (2012). Ki-67 predicts disease recurrence and poor prognosis in pancreatic neuroendocrine neoplasms. Surgery 152:107

CXCR4^{+}: Marchesi, F, Monti, P, *et al* (2004). Increased survival, proliferation, and migration in metastatic human pancreatic tumor cells expressing functional CXCR4.
Cancer Res 64:8420

DPP4(CD26)$^{++}$: Yan, L, Tian, X, *et al* (2020). CD26 as a Promising Biomarker for Predicting Prognosis in Patients with Pancreatic Tumors. Onco Targets Ther 13:12615

SARS-CoV-2

Thy-1 (ECs/MSC): Hayashi, T, Sano, K, *et al* (2022). Pathological Evidence for Residual SARS-CoV-2 in the Micrometastatic Niche of a Patient with Ovarian Cancer. Curr Issues Mol Biol. 44:5879
Valeri, A, Chiricosta, L, *et al* (2021). SARS-CoV-2 Exposed Mesenchymal Stromal Cell from Congenital Pulmonary Airway Malformations: Transcriptomic Analysis and the Expression of Immunomodulatory Genes. Int J Mol Sci 22:11814

Hepcidin$^+$ iron down: Liu, W, Zhang, S, *et al* (2020). Depriving Iron Supply to the Virus Represents a Promising Adjuvant Therapeutic Against Viral Survival. Curr Clin Microbiol Rep 7:13

Peng, D, Gao, Y, *et al* (2022). The Relationship Between Hepcidin-Mediated Iron Dysmetabolism and COVID-19 Severity: A Meta-Analysis. Front Public Health 10:881412

CD44$^+$: Albtoush, N, Petrey, A. C. (2022). The role of hyaluronan synthesis and degradation in the critical respiratory illness COVID-19. Am J Physiol Cell Physiol 322:C1037

CD24$^+$ treat: Song, N. J, Allen, C, *et al* (2022). Treatment with soluble CD24 attenuates COVID-19-associated systemic immunopathology. J Hematol Oncol 15:5

CTNNB1^{hi}: Chatterjee, S, Keshry, S. S, *et al* (2022). Versatile beta-Catenin Is Crucial for SARS-CoV-2 Infection 10:e0167022

Hazra, S, Chaudhuri, A. G, *et al* (2022). Integrated network-based multiple computational analyses for identification of co-expressed candidate genes associated with neurological manifestations of COVID-19. Sci Rep 12:17141

HMGB1$^+$ serum: Gowda, P, Patrick, S, *et al* (2021). Glycyrrhizin prevents SARS-CoV-2 S1 and Orf3a induced high mobility group box 1 (HMGB1) release and inhibits viral replication. Cytokine 142:155496

Ki67$^+$ (T cells): Sattler, A, Angermair, S, *et al* (2020). SARS-CoV-2-specific T cell responses and correlations with COVID-19 patient predisposition. J Clin Invest 130:6477

Mann, E. R.Menon, M, *et al* (2020). Longitudinal immune profiling reveals key myeloid signatures associated with COVID-19. Sci Immunol 5:eabod6197

CXCR4$^+$: Niemann, B, Puleo, A, *et al* (2022). Biologic Functions of Hydroxychloroquine in Disease: From COVID-19 to Cancer. Pharmaceutics 14:2551

DPP4^{++}: Bassendine, M. F, Bridge, S. H, *et al* (2020). COVID-19 and comorbidities: A role for dipeptidyl peptidase 4 (DPP4) in disease severity?. J Diabetes 12:649

SLE (Systemic Lupus Erythematosus)

MSC (CD90,CD105, CD73) in SLE: Dong,L, Pu.Y, *et al* (2018). Human umbilical cord mesenchmal stem cell-derived extracellular vesicles promote lung adenocarcinoma growth by transferring miR-410. Cell Death Dis 9:218

Hepcidin$^+$ Iron$^-$ in SLE: Kunireddy, N., Jacob, R. *et al* (2018). Hepcidin and Ferritin: Important mediators in inflammation associated anemia in systemic lupus erythematosus patients. Indian J Clin Biochem 33:406

CD44^{hi} in SLE: Yung,S., Chan, T.M. (2012). The role of hyaluronan and CD44 in the pathogenesis of lupus nephritis. Autoimmune Dis 2012:207190

CD24^{hi} in SLE: Blair, P.A., Norena, L.Y. *et al* (2010). CD19(+)CD24(hi)CD38(hi) B cells exhibit requlatory capacity in healthy individuals but are functionally impaired in systemic lupus erythematosus patients. Immunity 32:129

Urokinase-CTNNB1$^+$ in SLE: Zhuang, Y., Zhang, X., *et al* (2022). Exploring the molecular mechanism of Zhi Bai Di Huang Wan in the treatment of systemic lupus erythematosus based on network pharmacology and molecular docking techniques. Processes 10:1914

HMGB1$^+$ in SLE serum: Liu, T., Son, M, Diamond, B., (2020). HMGB1 in systemic lupus erythematosus. Front Immunol 11:1057

Ki67$^+$ in NK cells in SLE: Hudspeth,K., Want,S., *et al* (2019). Natural killer cell expression of Ki67 is associated with elevated serum IL-15, disease activity and nephritis in systemic lupus erythematosus. Clin Exp Immunol 196:226

CTNNB1$^+$ in SLE: Zhuang, Y., Zhang, X. *et al* (2022). Exploring the molecular mechanism of Zhi Bai Di Huang Wan in the treatment of systemic lupus erythematosus based on network pharmacology and molecular docking techniques. Processes 10:1914

DPP4 (CD26) in SLE: Huang, J., Liu, X., *et al* (2022). Emerging role of dipeptidyl peptidase-4 in autoimmune disease. Frontiers in immunology 13:830863

CD5 decreased in SLE: Omar, H.H., Nasef, S.I., *et al* (2017). CD5+ B lymphocytes in systemic lupus erythematosus patients: relation to disease activity. Clin Rheumatol 36:2719

CD1d dcrease in SLE: Bosma,A., Abdel-Gadir,A., *et al* (2012). Lipid-antigen presentation by CD1d(+) B cells is essential for the maintenance of invariant natural killer T cells. Immunity 36: 477

CD22^{h} in SLE: CD22: Clark, E.A., Giltiay, N.V. (2018). A regulator of innate and adaptive B cell responses and autoimmunity. Front Immunol 9: 2235

CD22^{++}CD11b^{++} in SLE: Ding,C., Ma, Y., *et al* (2013). Intergrin CD11b negatively regulates BCR signaling to maintain autoreactive B cell tolerance. Nat Commun 4:2813

AID^{+} in SLE-IgG: Kumar, R., DiMenna, L.J., *et al* (2014). Biological function of activation-induced cytidine deaminase (AID). Biomed J 37:269

CD180^{-} in SLE: You, M., dong, G., *et al* (2017). Ligation of CD180 inhibits IFN-alpha signaling in a lyn-PI3K-BTK-dependent manner in B cells. Cell Mol Immunol 14:192

CD21$^{-/low}$CD27^{-} in SLE: Horisberger, A., Humbel,M., *et al* (2022). Measurement of circulating CD21(-)CD27(-) B lymphocytes in SLE patients is associated with disease activity independently of conventional serological biomarkers. Sci Rep 12:9189

T-bet^{hi}CD11c^{hi} in SLE: Wang,S., Wang, J., *et al* (2018). IL-21 drives expansion and plasma cell differentiation of autoreactive CD11c(hi)T-bet(+) B cells in SLE. Nat Commun 9:1758

TCR7^{+}TCR9^{+} in SLE: Wen, L., Zhang, B., *et al* (2023). Toll-like receptors 7 and 9 regulate the proliferation and differentiation of B cells in systemic lupus erythematosus. Front Immunol 14:1093208
